# Strong phenotypic plasticity limits potential for evolutionary responses to climate change

**DOI:** 10.1038/s41467-018-03384-9

**Published:** 2018-03-08

**Authors:** Vicencio Oostra, Marjo Saastamoinen, Bas J. Zwaan, Christopher W. Wheat

**Affiliations:** 1 Department of Genetics, Evolution and Environment, University College London, The Darwin Building, Gower Street, London, WC1E 6BT UK; 2 Department of Plant Sciences, Laboratory of Genetics, Wageningen University, PO Box 16, 6700AA Wageningen, The Netherlands; 3 Organismal and Evolutionary Biology Research Programme, University of Helsinki, PO Box 65, Helsinki, FI-00014 Finland; 4 Department of Zoology, Population Genetics, Stockholm University, S-10691 Stockholm, Sweden

## Abstract

Phenotypic plasticity, the expression of multiple phenotypes from one genome, is a widespread adaptation to short-term environmental fluctuations, but whether it facilitates evolutionary adaptation to climate change remains contentious. Here, we investigate seasonal plasticity and adaptive potential in an Afrotropical butterfly expressing distinct phenotypes in dry and wet seasons. We assess the transcriptional architecture of plasticity in a full-factorial analysis of heritable and environmental effects across 72 individuals, and reveal pervasive gene expression differences between the seasonal phenotypes. Strikingly, intra-population genetic variation for plasticity is largely absent, consistent with specialisation to a particular environmental cue reliably predicting seasonal transitions. Under climate change, deteriorating accuracy of predictive cues will likely aggravate maladaptive phenotype-environment mismatches and increase selective pressures on reaction norms. However, the observed paucity of genetic variation for plasticity limits evolutionary responses, potentially weakening prospects for population persistence. Thus, seasonally plastic species may be especially vulnerable to climate change.

## Abstract (web summary)

Phenotypic plasticity—the ability to express multiple phenotypes from the same genome—is a widespread adaptation to environmental variability. Here, Oostra et al analyze transcriptomes of an African butterfly with distinct seasonal phenotypes, and observe lack of variation for plasticity, limiting potential for evolutionary responses to climate change.

## Introduction

Understanding how populations adapt to changing environments is of fundamental importance for assessing their evolutionary and ecological dynamics, and for predicting population resilience to climate change. Phenotypic plasticity, the expression of different phenotypes from the same genome in response to environmental variation, is a widespread adaptation in seasonal habitats that allows organisms to maximise fitness as they track the predictable cycles of contrasting ecological conditions^1^. These cycles place divergent selective pressures on an organism’s life history, rewarding phenotype-environment matching via seasonally plastic strategies, such as timing of breeding in birds, the diapause decision in multivoltine insects, and seasonal wing patterns in butterflies^2^.

While the ecological relevance of phenotypic plasticity is evident, its on-going impact on evolutionary dynamics is more contentious^3–6^. To what extent and under what circumstances phenotypic plasticity potentiates evolutionary adaptation to novel environments and thus facilitates population persistence under environmental change, or instead limits such adaptation, remains unresolved. This question has particular urgency in the context of rapid climate change, which is already having profound impacts on biodiversity and ecosystem functioning^7–10^. Unfortunately, the integration of biological mechanisms into predictive models of resilience to climate change is hampered by a lack of empirical data, for crucial parameters, including physiology, phenology and genetic variances^10^.

An important limit on adaptive potential in seasonal habitats is the lack of genetic variation for plasticity^9, 11^. Theoretical models of plasticity and population resilience generally assume such variation to be sufficiently present (e.g.,^11, 12^), but there is actually little empirical support for this assumption, as data on natural genetic variation for seasonal plasticity in natural populations is scarce (e.g.,^13^ but see e.g.,^14–16^). Of concern is the possibility that in predictable environments, selection against phenotype-environment mismatches will deplete variation in plasticity. In seasonally plastic organisms there is strong selection favouring a match between the expressed phenotype and what is needed to survive and/or reproduce in the prevailing seasonal environment. This requires predictability of seasonal transitions, and tuning of the phenotypic response for a particular trait (the reaction norm) to the reliability of environmental cues that predict the selective environment^17, 18^. If a cue is highly reliable, selection favours a trait reaction norm that is highly sensitive to the cue. Alternative reaction norms for that trait, that are less sensitive to the reliable cue, or tuned to a different, unreliable cue, will produce a maladaptive mismatch between phenotype and environment^2, 18^. Ongoing purifying selection against such mismatches will, under stable long-term climatic conditions, remove standing genetic variation for plasticity from the population.

Under climate change, characterised by increased environmental stochasticity^19^, the reliability of existing environmental cues as predictors for seasonal progression will likely diminish. As a consequence, the most common (i.e., previously adaptive) reaction norm is then more likely to produce mismatches between phenotype and environment. Such maladaptive phenological shifts are already being observed in many species^20, 21^. Under these conditions, selection will favour alternative reaction norms, for example with a different sensitivity to the environmental cue or bet-hedging strategies. Theoretical models predict that reduced environmental predictability places species with strong plasticity under increased extinction risk^18, 22^, and a rapid response to selection for alternative reaction norms hinges on the presence of sufficient standing variation for plasticity. However, if past purifying selection has depleted this variation, the potential for evolutionary change is limited in the short term^23^, reducing the probability of population persistence under rapid climate change^18, 22^. Thus, rather than giving rise to adaptable generalists, phenotypic plasticity in seasonal habitats may generally result in specialists with reduced short-term adaptive potential that are particularly vulnerable to climate change. While selection on plasticity for timing of breeding has been documented in a wild-bird population in response to climate change^14^, many other species or populations lack such adaptive potential^13^, raising unanswered questions about why such variation may be depleted.

**Fig. 1:**
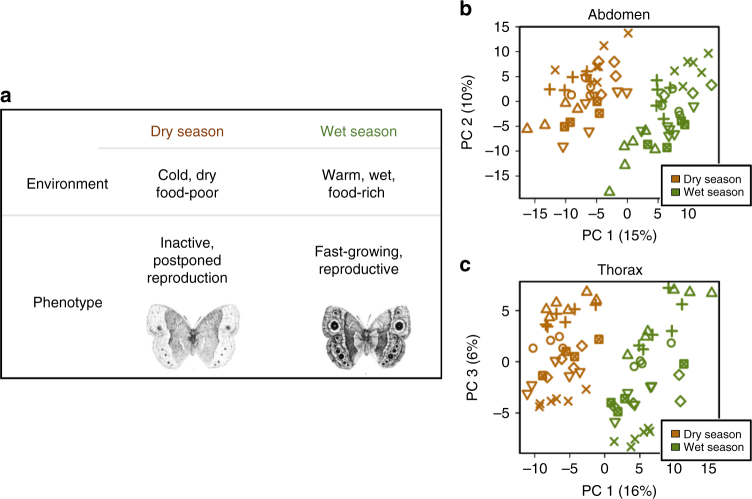
Pervasive seasonal plasticity and intra-population genetic variation across the transcriptome. **a** *Bicyclus anynana* butterflies of the dry (brown) and wet (green) season differ in a suite of behavioural, life history, and morphological traits, depending on the seasonal environment in which they developed. **b**, **c** Principal components analysis (PCA) of whole-transcriptome expression profiles for abdomen (**b**) and thorax (**c**) significantly separates individuals reared in wet (green) or dry (brown) season conditions along the first Principal Component (accounting for 15–16% of variance). For abdomen, the second PC (10% of variance) significantly separates individuals from different full-sib families (indicated by different symbols), whereas for thorax the third PC (6% of variance) separates families. Families are also significantly separated by several other PCs, together accounting for 56 and 27% of variance in abdomen and thorax (see Supplementary Figs. 5 and 6 for all PCs and statistical tests). Butterfly drawings © Clara Lacy

In order to clarify the role of plasticity in adaptation to climate change, we analyse adaptive potential in a textbook model of seasonal plasticity: the African savannah butterfly *Bicyclus anynana*^24^ (Fig. 1a). This species produces generations of butterflies that are highly distinctive in wing pattern, behaviour, and life history strategy across alternating seasonal environments^25–27^. As life histories are highly relevant for climate adaptation^9^, our focus here is on seasonal plasticity in life history. In the warm wet season, when food and reproductive opportunities are abundant, these butterflies live short lives of fast growth and maximal reproduction, allocating less resources to body maintenance. In contrast, in the cool dry season, when adult food (fruit) is limited and larval food (grasses) absent, these adults express a life history focused on inactivity, postponed reproduction and long lifespan^26^. The resulting life history syndrome comprises an integrated suite of coordinated traits, responding in a threshold-like manner to the seasonal environment via a common temperature-sensitive hormonal regulator^28, 29^.

Encoded by a shared genome deployed differently across the seasons, such alternative life histories are expected to be regulated by distinct transcriptional programmes. Here we focus on thorax and abdomen tissues, as we previously discovered that their relative importance, such as in reproduction (abdomen) and flight performance (thorax), is season-dependent^30, 31^, and they likely express genes relevant for lifespan, metabolism and stress resistance. Our study thus enables identifying tissue-specific and systemic mechanisms involved in plasticity. Furthermore, analysing the transcriptome as a highly dimensional plastic phenotype provides a comprehensive and quantitative picture of plasticity and its evolutionary potential. For wing pattern the potential for plasticity to evolve has been found to be limited^32^, but whether life history traits are similarly constrained is unknown. Finally, to further elucidate adaptive potential in plasticity, we additionally analyse the effect of stress, which has been suggested to either aid or constrain adaptive potential^33–35^. In a previous phenotypic study in *Bicyclus* butterflies, we found substantial effects of food stress on all five life history traits studied, with two showing season-specific responses, and for one trait the heritability was affected by food stress^33–36^. Given that phenotypic studies can only feasibly analyse a handful of traits, our study here aims to contribute to a more comprehensive, quantitative understanding of the role of stress in adaptive potential.

Here we quantify the potential for evolutionary change in plasticity, combining an RNA-Seq approach with a full-factorial split-brood design across 144 individual transcriptomes. Specifically, we (1) characterise the transcriptional architecture of the seasonal phenotypes, (2) quantify intra-population genetic variation for plasticity, (3) assess footprints of selection on plasticity in coding regions and (4) determine the role of stress in constraining or increasing adaptive potential. Our analysis uncovers high amounts of seasonal plasticity in the transcriptome, reflecting a genome-wide plasticity programme, consistent with the integrated suite of traits involved in the seasonal adaptation. Strikingly, intra-population genetic variation for this plastic response is largely absent, with low levels of gene-by-environment interaction and highly similar seasonal responses across families. We hypothesise that this reduction reflects strong purifying selection in the savannah habitat where temperature serves as a reliable cue for predicting seasonal transitions, and the fitness cost of a mismatched phenotype is high^37^. Consistent with this, Tajima’s D, a measure of DNA sequence polymorphism, is reduced in genes differing in plasticity across families, while this gene set shows no such reduction in an outgroup species lacking strong polyphenism. Stressful conditions during development decrease the transcriptional divergence between the seasons, but this is not accompanied by increased genetic variance for plasticity. Our results show that lack of genetic variation for plasticity may critically limit evolutionary potential and population persistence under environmental change, providing empirical support for theoretical predictions that species with strong phenotypic plasticity may be at elevated extinction risk when environmental predictability breaks down.

## Results

### Seasonal plasticity across the transcriptome

In order to understand how the shared genome is deployed differently across the seasonal environments to produce the distinct phenotypic morphs, we analysed the transcriptional architecture of plasticity. We compared gene expression between the wet and the dry season forms, in a full-factorial analysis of environmental and heritable effects across 72 individuals, analysing thorax and abdomen separately. Three independent analyses revealed large fractions of the abdomen and thorax transcriptome to be involved in seasonal plasticity, consistent with the large effect of the seasonal environment on many life history phenotypes^28, 36^. Differential expression analyses revealed that in abdomen and thorax, 46 and 47% of genes showed significant season-biased expression, respectively (FDR < 0.05; Supplementary Fig. 1), with alternative mapping and filtering approaches yielding similar results (Supplementary Figs. 2–4; Supplementary Table 1). Principal components analysis (PCA) of the whole-transcriptome expression profiles found PC1, accounting for 15–16% of total variance, significantly separating individuals from wet and dry season environments (two-way ANOVA *F*_1,55_ > 38, FDR < 10^−6^; Fig. 1; Supplementary Figs. 5, 6). Hierarchical clustering of gene expression confirmed these patterns, with individuals clustering strongly by season (Supplementary Fig. 7).

**Fig. 2:**
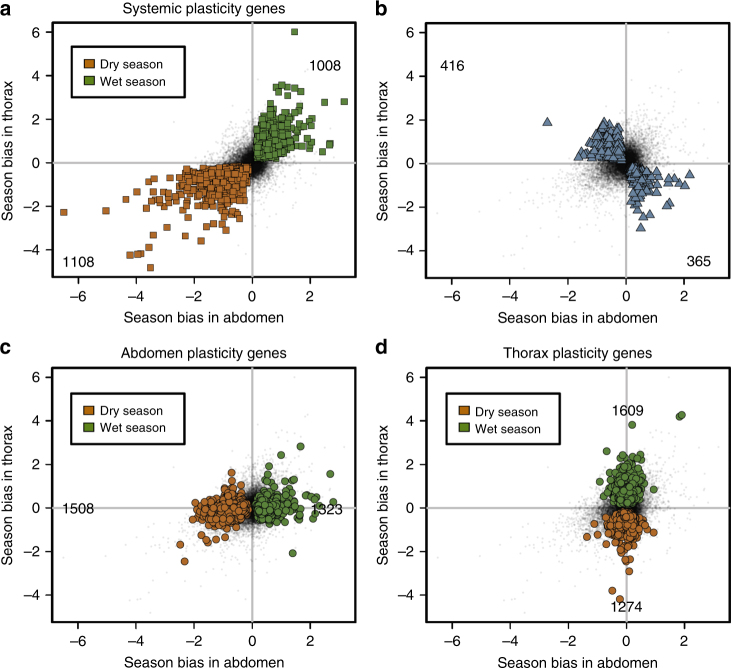
Systemic and tissue-specific components of the plasticity programme. For each gene expressed in both body parts, seasonal expression bias (log_2_ fold change of expression in wet season compared to dry season) in thorax (vertical axis) is plotted against expression bias in abdomen (horizontal axis), with positive fold change values indicating higher expression in wet season, and negative values indicating higher expression in dry season. Each gene is represented by a black dot, and genes significantly differentially expressed (FDR < 0.05) between wet and dry season are coloured in green and brown for wet and dry season-biased genes, respectively (**a**,** c**, **d**), or in blue (**b**). Numbers in each quadrant indicate numbers of significant wet or dry season-biased genes. **a** The systemic plasticity programme is represented by 2115 genes that show the same response to the seasonal environment in both body parts, representing 14 and 17% of the abdomen and thorax transcriptome, respectively, and enriched for 89 functional GO terms. **b** Expression for 781 genes shows opposite patterns of season bias between the two body parts, i.e., expression is wet season-biased in one body part and dry season-biased in the other body part. These genes are enriched for 37 GO terms. **c**, **d** The abdomen- and thorax-specific plasticity programmes are represented by 32 and 30% of genes in each body part, and are enriched for 155 and 189 GO terms. This includes genes expressed in both body parts but season-biased in only one (presented in this figure), as well as genes expressed exclusively in one body part (Supplementary Notes). Selected GO terms are visualised in Supplementary Fig. 8, all GO terms are listed in Supplementary Data 2, and a list of all season-biased gene is presented in Supplementary Data 1

Examining the seasonal plasticity programme in more detail revealed systemic and tissue-specific components, both at the level of individual genes and functional processes. A total of 2115 genes showed the same response to the seasonal environment in both body parts, representing 14 and 17% of the abdomen and thorax transcriptome (Supplementary Data 1), respectively, and these were enriched for 89 functional GO terms. In addition to these genes showing systemic plasticity, 32 and 30% of genes showed tissue-specific seasonal bias in abdomen and thorax transcriptomes, and were enriched for 155 and 186 GO terms, of which 109 and 140 were unique in each body part, and 37 were shared with concordant seasonal bias (Fig. 2; Supplementary Data 2). As expected for plasticity genes involved in the seasonal phenotypes, average expression levels for season-biased genes were significantly higher than for unbiased genes (Supplementary Fig. 9). Thus, within and across tissues, the seasonal environment is a major determinant of transcriptional variation, representing a genome-wide seasonal plasticity programme. Both the large numbers of genes involved in plasticity and their functions are consistent with the broad suite of life history traits involved in the seasonal adaptation^28, 36^. While a substantial part of the transcriptional response is systemic, reflecting an integrated and coordinated environmental response across the body, the largest component to the seasonal transcriptional response is tissue-specific, reflecting modular, independent responses to the seasonal environment (Supplementary Notes).

### Lack of genetic variation for plasticity

The extent to which the transcriptional plasticity programme can evolve critically depends on standing genetic variation that modulates plasticity in gene expression. Based on five different analyses, we found that this variation is reduced in the population, contrasting starkly with the high levels of standing variation for average gene expression.

**Fig. 3:**
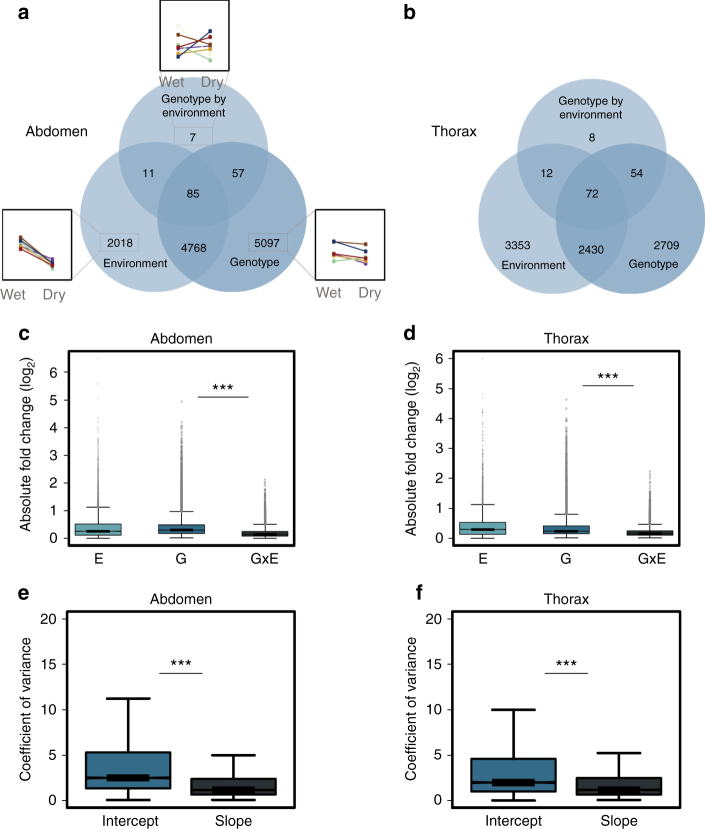
Large-scale reduction of genetic variation for seasonal plasticity across the transcriptome. **a**, **b** An order of magnitude more genes show significant differential expression due to seasonal environment and genetic background than due to the interaction between environment and genetic background for abdomen (**a**) and thorax (**b**). Within each Venn diagram, numbers of differentially expressed genes (FDR < 0.05) are indicated for seasonal environment (left), genetic background (right), and their interaction (top), as well as overlap in responses among genes. Small insets in **a** illustrate expression patterns for each gene group, showing mean normalised expression in wet and dry season for random genes in each group, with reaction norms for different full-sib families represented by different colours. **c**, **d** Across all genes in the abdomen (**c**) and thorax (**d**) transcriptome, mean absolute expression changes (log_2_ fold change) due to the interaction between environment and genetic background (i.e., transcriptional plasticity or GxE; right boxplot) are 43–51% smaller than those due to genetic background (i.e., genetic variation for average expression or G; middle boxplot; ***P < 0.0001). Only 120 and 88 transcripts (in abdomen and thorax, respectively) showed more than a twofold expression change as a result of inter-family differences in plasticity, compared to 1054 and 766 transcripts above that threshold for inter-family difference in average expression. **e**, **f** Families are more similar in how expression responds to the environment than in average expression levels, with reaction norms for expression of all genes showing on average 57 and 32% lower across-family variance in slope (right, black) than in intercept (left, blue) for abdomen (**e**) and thorax (**f**), respectively (***P < 0.0001). *P*-values are for two-sample Wilcoxon signed-rank tests (two-sided). Upper whiskers are at the upper quartile plus 1.5× the interquartile range or at the maximum value (whichever is lowest), whereas lower whiskers extend to the lower quartile minus 1.5× the interquartile range or to the minimum value (whichever is highest)

First, expression of only 1% of genes (160 and 146 genes in abdomens and thoraces, respectively) was significantly affected (FDR < 0.05) by the interaction between seasonal environment and family, i.e., by a genotype-by-environment interaction (GxE; see Supplementary Data 3 for a list of all GxE genes). In contrast, genetic background (i.e., family) significantly affected average expression for 66% and 42% of genes in abdomen and thorax, respectively (Fig. 3a, b; Supplementary Fig. 1). Using alternative filtering and mapping approaches or applying an additional fold change >2 threshold yielded very similar results (Supplementary Notes; Supplementary Figs. 2–4; Supplementary Table 1).

Second, across all genes (irrespective of being differentially expressed), the absolute expression changes between families in transcriptional plasticity were 43–51% smaller than those between families in average expression (Fig. 3c, d; two-sample Wilcoxon signed-rank tests *Z* < −133, *P* < 10^−16^). Only 120 and 88 transcripts (in abdomen and thorax, respectively) showed more than a twofold expression change as a result of inter-family differences in expression plasticity, compared to 1054 and 766 transcripts above that threshold for inter-family difference in average expression.

Third, PCA confirmed that inter-family variation in plasticity of expression represents a tiny fraction of total transcriptional variance, contrasting with variation between families being a large driver of average gene expression. In thoraces, none of the first 13 Principal Components (together accounting for 62% of total variance) associated with the interaction between seasonal environment and genetic background (two-way ANOVA, FDR > 0.49, *F* < 2.0), while in abdomen only PC 13 (accounting for 1.5% of total variance) was significantly affected by the interaction between seasonal environment and genetic background (FDR = 0.03, *F* = 3.9). In contrast, a total of 7 and 9 PCs significantly associated with genetic background as main effect, in abdomen and thorax respectively, and together these PCs explained 56 and 27% of total transcriptional variance in each body part (two-way ANOVAs; FDR = 0.01–10^−24^; *F* = 3.3–85.1; Supplementary Figs. 5, 6).

Fourth, analysis of reaction norms in gene expression revealed that families are much more similar in how expression responds to the environment than in their average expression levels. We computed family-specific seasonal reaction norms, comparing across-family variance in slope (plasticity) and intercept (average expression), and found that variance in slope was 57 and 32% lower than variance in intercept, for abdomen and thorax respectively (Fig. 3e, f; *Z* > 36, *P* < 10^−16^).

Finally, we observed high-positive genetic correlations in gene expression across seasonal environments (Supplementary Fig. 10), with median Spearman Rank correlations of +0.67 and +0.47, for abdomen and thorax, respectively. This indicates that for many genes, expression in one season is genetically coupled with expression in the other season, and there is limited genetic variation for expression that is independent between the seasons.

Together, these analyses provide evidence for a substantial reduction of intra-population genetic variation for plasticity, with low levels of gene-by-environment interaction and different full-sib families having highly similar seasonal responses. This contrasts sharply with the high levels of genetic variation for expression averaged across the seasons, where families vary extensively in average gene expression patterns. As a consequence, there is ample opportunity for evolutionary change in average gene expression in the same direction in both seasons, but potential for change in plasticity is limited.

### Footprints of selection on plasticity in the transcriptome

In order to assess whether the observed lack of genetic variation for plasticity is due to purifying selection on reaction norms, we analysed polymorphism in coding sequence of abdomen-expressed genes. We compared genes that show inter-family differences in plasticity (i.e., gene-by-environment interaction or GxE) with those showing no GxE. Specifically, we quantified Tajima’s D, the difference between the fraction of pairwise nucleotide differences *π* and fraction of segregating sites *θ*^38^, and found a significant decrease to a median of 0.32 for genes showing GxE (*n* = 158) from 0.40 for genes showing no GxE (*n* = 14,749; Mann–Whitney *P* = 0.041). This decreased Tajima’s D, or excess of rare alleles, was not observed for other gene repertoires (Supplementary Fig. 11). While negative values of Tajima’s D can indicate positive selection, genes experiencing positive selection are expected to be rare in the genome and outliers compared to genome-wide averages. Instead, our observed reduction in Tajima’s D among the GxE genes suggests increased selection against novel variants (cf.^39^), consistent with increased purifying selection on these reaction norms.

To further assess whether other potential processes than purifying selection could be at work, we considered whether novel genes might be driving the observed polymorphism pattern, as duplicated genes that have recently become fixed in the population can also show reduced Tajima’s D, i.e., an excess of rare alleles^40^. However, this alternative explanation is unlikely for several reasons. First, recently fixed novel genes should also show reduced pairwise diversity (*π*)^40^, but instead we observe similar levels of *π* between genes with inter-family variation in plasticity (GxE) compared to the rest of the genome (Supplementary Fig. 12). Second, these genes do not show significant enrichment for novel, lineage-specific genes. To assess this, we searched for orthologs in *Pieris rapae*, a temperate butterfly species that shows much lower levels of seasonal polyphenism, and defined novel genes as those for which we could not find any putative ortholog (although these genes could also represent gene loss in the *P. rapae* lineage). Of the 10,466 *B. anynana* transcripts with a *P. rapae* hit, 1.0% (101) showed gene-by-environment interaction in expression. Among the 4583 *B. anynana* transcripts without a hit to *P. rapae*, this proportion was slightly higher, with 1.3% (59) of these *B. anynana* transcripts showing GxE in expression. However, this was not significant (*χ*^2^ = 2.8, *P* = 0.09), making it unlikely that the low level of Tajima’s D among the GxE genes is driven by these genes being more novel than other genes in the genome. Finally, if purifying selection in a highly predictable seasonal environment caused the observed reduction in Tajima’s D for genes showing GxE, these genes should show no such reduction in a species without the specific selective history of *B. anynana*. Using population re-sequencing data for *P*. *rapae*, we assessed Tajima’s D in the orthologs of the *B. anynana* GxE genes, and found no significant difference compared to the rest of the genome (Mann–Whitney *P* = 0.32; Supplementary Fig. 13a). However, in *P*. *rapae* pairwise nucleotide polymorphism (*π*) in these genes was 43% higher than the rest of the genome (*P* < 0.005), suggesting that in this species this set of genes might be under relaxed selection (Supplementary Fig. 13b). In conclusion, our findings suggest that the most likely explanation for the lack of gene-by-environment interactions and excess of rare alleles at GxE genes in *B. anynana* is purifying selection in its natural habitat, the highly predictable savannah environment.

### Developmental food stress and adaptive potential

We assessed whether stressful conditions might aid adaptive potential, either by inducing phenotypic expression of genetic variance that is not visible under normal conditions, or by disrupting normal patterns of plasticity. We exposed developing larvae (of both seasonal forms) to a period of food stress, and observed only minor effects on seasonal plasticity (Supplementary Notes; Supplementary Fig. 1; Supplementary Table 2). Expression of <10 genes showed a significant interaction between seasonal plasticity and food stress, indicating that for virtually all genes the seasonal response was not different under stressed compared to ad libitum conditions. Examining the effect of food stress in more detail for specific gene repertoires revealed subtle stress-induced shifts in normal seasonal expression patterns. In particular, stress pushed the typical dry season morph towards a slightly more wet season-like transcriptional profile, partly driven by an emergency response in the abdomen comparable to a terminal reproductive investment, with wet season genes showing increased expression upon stress (Supplementary Notes; Supplementary Fig. 14). However, this limited reduction in transcriptional divergence between the seasonal forms was not accompanied by increased genetic variance for plasticity. The three-way interaction between genetic background, food stress treatment and seasonal environment only affected a small number of genes, indicating a very limited effect of stress on genetic variation for plasticity and thus potential for stress to release genetic variation in plasticity. These results were consistent when using alternative filtering and mapping approaches or applying an additional fold change >2 threshold (Supplementary Figs. 2–4; Supplementary Table 1). In conclusion, we found no evidence that stressful conditions released genetic variation.

## Discussion

The role plasticity plays in enhancing or constraining adaptive potential has been discussed extensively in the literature (e.g.,^3–6, 8, 12^). On the one hand, evolutionary adaptation to novel environments may be constrained due to plasticity reducing the strength of selection by buffering environmental variation and thus hiding low-fitness variants from selection. On the other hand, plasticity could facilitate evolutionary adaptation in novel environments as plastic populations express a wider range of phenotypes than non-plastic populations, and new phenotypic optima may be more easily reached if they can be directly induced by the environment rather than produced from new genetic variants). More recent theoretical studies highlight the importance of environmental predictability in determining whether strong plasticity is an advantage or liability under rapid environmental change^18, 22^, and suggest that a reduction in environmental predictability places species with strong plasticity under increased extinction risk.

Here we study adaptive potential in a seasonally plastic butterfly with highly distinct alternative phenotypes, and observe a striking lack of intra-population genetic variation for plasticity. Families display very similar transcriptional responses to the seasonal environment for nearly all transcripts, with significantly reduced inter-family variance in reaction norm slope. In marked contrast, families differ significantly in average expression for approximately half of all transcripts, with high inter-family variance in reaction norm intercept. These high levels of variation for average gene expression have also been observed in other animal populations (e.g., in fruitflies^41^; butterflies^15^; sticklebacks^16^; and other species in the wild^42^). The observed lack of variation for plasticity is not due to inbreeding, as that would also depress genetic variation for average expression among families. Another potential confounder, relaxed purifying selection due to laboratory breeding conditions, is also unlikely to play an important role. Relaxed selection on plasticity would result in increased rather than decreased genetic variation for plasticity^43^ and potentially reduced plasticity overall (cf.^44^). Instead, our results suggest that the most likely explanation is enhanced purifying selection solely upon reaction norms, consistent with variation in expression plasticity for these genes being largely absent in the founding population.

*Bicyclus anynana* inhabits a strongly seasonal savannah environment, with vastly differing food availability and reproductive opportunities between the dry and wet seasons, making it crucial to produce the right phenotype at the right moment. In Nkhata Bay in Malawi, source of the laboratory population^24^, transitions between the seasons are highly predictable, with a strong positive correlation between monthly averaged precipitation and minimum temperature in the previous month^45^ (*ρ*_pearson_ = +0.85; Supplementary Fig. 15). This puts a premium on a single-reaction norm optimally tuned to the specific temperature cue predicting seasonal transitions. Alternative reaction norms, producing the wrong phenotype, would lead to a maladaptive mismatch between seasonal environment and expressed phenotype^37^. Given the vastly contrasting selective pressures across the seasons, reaction norms producing such mismatches would likely be strongly selected against. This ongoing purifying selection should deplete genetic variation for plasticity, without affecting overall levels of genetic variation for expression. Consistent with this, we find relatively few genes showing gene-by-environment interaction in expression, and those that do show GxE have an excess of rare mutations (i.e., low Tajima’s D), consistent with increased purifying selection. Additional analyses of these GxE genes revealed that they neither exhibit patterns of pairwise diversity consistent with recent gene duplication events^40^, nor are enriched for novel genes. Finally, we assessed the patterns of genetic variation in the orthologs of these GxE genes in *P*. *rapae*, a temperate butterfly species without strong seasonal polyphenism, and found them to be more polymorphic than the rest of the genome, suggestive of a relaxation of purifying selection. This latter finding is consistent with considerable to high levels of gene-by-environment interaction in the transcriptome among many species that lack seasonal polyphenism, including *Drosophila*^46^, *Caenorhabditis elegans*^47^, yeast^48^, butterflies^15^, sticklebacks^16^ and other fish^49^. However, not all species show these patterns, and they vary with the type of environmental treatment and whether laboratory or wild populations were assayed (e.g.^50^). Unfortunately, most studies focus on genetic variation in plasticity across multiple populations (but see refs. ^15, 49^), and data on species in strongly seasonal environments is particularly lacking. Future analyses focused upon gene turnover and evolutionary rates within a phylogenetic framework, comparing species differing in seasonal and/or thermal plasticity, are likely to shed important light on the evolutionary forces acting on regulators of plasticity compared to the rest of the genome.

Our results on transcriptional plasticity support earlier work on phenotypic trait plasticity and evolution on this same population. Many seasonally plastic traits, such as growth rate, metabolic rate, starvation resistance and wing pattern also show significant intra-population genetic variation^31, 51–53^, and artificial selection was highly successful in shifting mean trait expression, i.e., the reaction norm intercept (e.g.,^52^). However, reaction norm slopes show high-across-environment genetic correlations and very limited genetic variation, precluding evolutionary responses to artificial selection targeting the slope of the reaction norm, at least in morphological traits^32^. Our transcriptional data, revealing largely absent GxE for gene expression in tissues involved in life history plasticity, together with phenotypic data where most traits lacked GxE^36^, strongly suggest that life history traits are similarly constrained (see also^51^).

The expression of genetic variance is environment-dependent, yet the impact of unfavourable environmental conditions on genetic variation, and hence adaptability under environmental change, is disputed^33, 35^. In our study, developmental food stress did not release genetic variance for plasticity, consistent with previous phenotypic observations in this species^36^ and in wild populations of other species^35^, indicating that stress does not necessarily aid adaptability under environmental change.

A large portion of the transcriptome is involved in seasonal plasticity, with almost half of all expressed genes showing season-biased expression. This is not surprising, given that the seasonal adaptation in *B. anynana* represents a broad and integrated life history syndrome, encompassing a suite of traits including growth rate, size at maturity, hormone physiology, metabolic rate, fat metabolism, reproductive strategy and starvation resistance^25, 26, 28, 36, 51, 52, 54^. Whereas some of these traits are likely underpinned by systemic transcriptional patterns, others such as reproduction are more compartmentalised into specific body parts and tissues. Indeed, we observe that the seasonal transcriptional response is partly driven by a systemic plasticity programme, at both the level of individual genes and functional pathways, and partly by tissue-specific regulation of gene expression. The simultaneous occurrence of both systemic and tissue-specific plasticity is consistent with the known regulation by ecdysteroid hormones of seasonal plasticity for a broad suite of wing pattern and life history traits. The hormone system enables integration and coordination of plasticity across the body via systemic hormone titres, while at the same time allowing flexibility and independence via compartmentalised, time- and tissue-specific responses^28, 29, 55, 56^. Fitting this model, season-biased genes for both abdomen and thorax were enriched for steroid signalling without overlap of individual genes, indicating tissue-specific roles for this hormone system. Genes involved in regulation of transcription (including DNA methylation) and in juvenile hormone signalling were also strongly season-biased in both body parts, underlining the importance of regulatory processes in seasonal plasticity. In addition, we also identified processes more directly linked to the phenotype, some of which were systemic, such as immunity, lipid metabolism and oxidoreductase activity, and others that were only enriched in the abdomen, such as response to oxidative stress (e.g., Catalase), or the thorax, such as processes related to the actin cytoskeleton, important for flight muscles. Interestingly, a substantial fraction (6%) of the shared transcriptome showed opposite patterns of season-bias between the tissues, with enrichment for processes like ubiquitination, cell division, lipid metabolism and translation. This likely reflects trade-offs between body parts, for example in investment in growth, storage and turnover of resources, which shift between seasons.

In comparison to our results for the abdomen and throax, a separate study in *B. anynana* found the seasonal forms to be much less differentiated in the head transcriptome^57^, suggesting that behavioural differences are less pronounced than physiological phenotypes, at least early in adult life. This underscores the modularity of seasonal plasticity, where body parts contribute specific functions to alternative morphs, a pattern often observed in other animals. For example in the beetle *Onthophagus taurus*, phenotypic plasticity is highly modular across the body, with different body parts showing distinct transcriptional responses and evolutionary rates^58^. Whether the modularity of plasticity observed in *B. anynana* enhances adaptive potential under environmental change remains to be tested.

Taken together, our results indicate that the potential for evolutionary change in plasticity in this population is constrained by the current lack of genetic variation for plasticity, at least in the short term, and any evolutionary response in plasticity will thus depend upon novel mutations affecting reaction norms. This limited adaptive potential will only surface if selective pressures change in such a way that the currently dominant reaction norm no longer has the highest fitness, and instead produces a mismatch between expressed phenotype and the new selective environment. Current climate change represents exactly such a scenario, as not only temperature means are increasing rapidly, but also variance in temperature and precipitation^19^. This will likely deteriorate the reliability of existing environmental cues for seasonal progression, increasing selective pressures for alternative reaction norms and, given the limited genetic variation for plasticity, weakening prospects for population persistence.

In many seasonal habitats, species depend on environmental cues such as temperature or day length to prepare for seasonal transitions^17^. A modelling study shows that decreased environmental predictability increases extinction risk for plastic species^22^, and indeed climate change, by affecting environmental cues, as well as seasonal timing, is already increasing mismatches between phenotype and seasonal environment^13, 14, 20, 21, 23^. Even where species respond to climate change by shifts in seasonal timing, the adjustment in phenology is often too subtle or too extreme, leading to frequent mismatches in phenotype with seasonal progression^21^. Although microevolutionary changes in such responses are crucial for population resilience to climate change, there is limited empirical data on the extent of natural genetic variation for seasonally plastic responses, curtailing our understanding of adaptive potential of populations facing climate change^8, 9, 23^. Our study illustrates that specialised seasonal plasticity may result in reduced adaptability in the face of environmental change via lack of genetic variation for seasonal reaction norms. Given the ubiquity of seasonal habitats across tropical and temperate areas, this likely applies to many species and thus represents an underappreciated limit to biotic climate change resilience.

## Methods

### Study organism and experimental design

We used a captive laboratory population of *B. anynana*, reared under standardised outbred conditions^24^. In order to assess the effect of genetic background, seasonal environment, food stress and their interactions on the transcriptome, we employed a full-factorial split brood design with 72 females from seven full-sib families, reared at two temperatures (19 °C corresponding to dry season, and 27 °C corresponding to wet season), and two developmental food stress treatments (ad libitum and food stress in the fifth larval instar). We chose a single sex (females) because for many phenotypic life history traits, sex interacts strongly both with seasonal plasticity and stress response^29, 36^. Within each family, the design was balanced with four families having three females for each combination of family, seasonal temperature and food treatment, and the three other families having two females per treatment group. This yielded sample sizes for the main effects of food, season and family of 36, 36 and 8–12, respectively, and 4–6 for the two-way family-by-food or family-by-season interactions (see Supplementary Tables 6 and 7 for details). These sample sizes are per tissue, as we sampled thorax and abdomen from each female, and analysed the transcriptomes separately. Sample sizes represent biological, not technical replicates, and were chosen to maximise the number of families while maintaining sufficient replication within families for each experimental factor. After sequencing, we removed five outliers (out of 144 samples), based on SNPs and expression clustering patterns. Within this same experiment, we have collected phenotypic data on additional individuals and families, which we published previously^36^. Larvae completed their full development in one of two temperature conditions representing the seasonal environments, while the developmental food stress treatments lasted two or 3 days during the fifth (last) larval instar^31, 36^, with control larvae receiving normal food (fresh maize leaves) and stressed larvae receiving no food, but agar only to avoid dehydration^31, 36^. Allocation of newly hatched first instar larvae to each of four experimental treatments was done randomly, with no blinding. One day after eclosion we sampled females for RNA isolation (snap-freezing in liquid N_2_), and separated thorax and abdomen for individual sequencing, using whole thorax and whole abdomen while removing heads, and all legs and wings.

### RNA sequencing and data pre-processing

We isolated RNA using TRIzol (Invitrogen) followed by the RNeasy Mini Kit (Qiagen) and a DNA digestion using the RNase-Free DNase enzyme (Qiagen). We sent total RNA for 144 samples (thorax and abdomen of 72 females) to BGI (People’s Republic of China) for mRNA purification, library preparation and sequencing (paired-end, 2 × 100 bp, mean insert size 350 bp, Illumina HiSeq 2000). We obtained a mean of 15.4 × 10^6^ raw PE 100 bp reads per sample (95% CI: 13–16 × 10^6^), totalling 2.2 × 10^9^ reads across 144 samples. We assessed quality using FastQC v. 0.10.1 (http://www.bioinformatics.babraham.ac.uk/projects/fastqc/) and trimmed reads using bbduk2 in bbmap v. 0.34.94 (http://bbmap.sourceforge.net/), trimming a mean of average 6.9% of reads per sample (95% CI: 4.5–10.7%). See Supplementary Data 4 for an overview of sequencing results for all libraries.

### De novo transcriptome assembly and annotation

Transcriptome assembly used Trinity v. 20140717^59^, combining reads from all libraries (i.e. thorax and abdomen), yielding 496,087 contigs. Because de novo transcriptome assemblies produce a large number of contigs per locus, resulting from transcriptional noise and misassemblies, as well as SNPs and isoforms, we collapsed our assembly to biologically meaningful transcripts by aligning the coding regions and keeping the longest transcript, using the EvidentialGene pipeline (http://arthropods.eugenes.org/EvidentialGene/trassembly.html). This yielded a filtered high-quality transcriptome of 35,747 contigs with an N50 of 1839 and N10 of 4561, with the vast majority being assembled near full length (Supplementary Fig. 16; Supplementary Table 4). To annotate it, we performed a blastp search of the 35,747 predicted proteins against the UniRef90 database^60^. After filtering (evalue <0.00001 and bitscore >50), this yielded valid UniRef protein names for 14,681 transcripts. Separately, we used Argot2 to obtain Gene Ontology (GO) terms for our transcriptome^61^, yielding 6107 unique GO terms for 15,991 transcripts, with a mean of 5.2 GO terms per annotated transcript. After removing GO terms with <4 or >500 genes, and as recommended^61^ terms with Argot2 total score <200, we retained 13,192 transcripts annotated with 2183 unique GO terms (3.4 terms per transcript).

### Mapping and transcript abundance estimation

We mapped trimmed reads against our filtered transcriptome using Bowtie2 v. 2.2.3^62^ allowing one mismatch between seed and reference, with an average of 83% reads mapped (95% CI: 72–93%; Supplementary Data 4). We quantified raw read counts per transcript per library using SAMtools idxstats^63^. To further reduce transcriptional noise prior to differential expression analysis, we applied a low-expression filter by removing genes with low or very limited expression, processing the 72 thorax and 72 abdomen libraries separately. We removed genes that were expressed in <3 samples, as well as genes with mean expression <0.25 counts per million (CPM). After filtering, we retained 15,049 and 12,567 genes for abdomen and thorax, respectively, accounting for >99.9% of all read counts.

### Robustness of results to filtering and mapping approaches

In order to exclude the possibility that our results would be biased by the choice of transcriptome filtering, mapping, transcript abundance estimation, or expression filtering strategy, we repeated differential expression analyses (a) without the low-expression filter, (b) using different reference transcriptome filtering, mapping and abundance estimation approaches, and (c) with an additional fold change 2 threshold for significant differential expression. In addition to the mapping approach described above, we used the mapping programs NextGenMap v0.5.0^64^ and RSEM v1.2.19^65^. The latter program (which uses Bowtie2 internally) was tested using both the unfiltered Trinity transcriptome assembly (at both isoform and gene level) and the Evigene-enriched transcriptome as a reference (see Supplementary Table 5 for details).

### Differential expression analysis

For thorax and abdomen separately, we performed differential expression analyses in edgeR v. 3.10.5^66^ to test the main effects of seasonal temperature, food treatment, family, the three two-way interactions, as well as the one three-way interaction between the main effects on expression of all expressed genes in the transcriptome (Supplementary Table 3). EdgeR incorporates methods to account for data heteroscedasticity. As input data for edgeR we used untransformed, raw count data (after filtering to remove genes with low expression, see above). Thus, expression of each individual transcript was modelled as follows: raw counts ~ seasonal temperature + food treatment + family + season-by-family + food-by-family + season-by-food + season-by-food-by-family. For each factor of interest, edgeR analyses yielded a fold change between the conditions, a likelihood ratio for the effect of that factor, and a corresponding *P-*value. We corrected these *P*-values for multiple comparisons using Benjamini and Hochberg’s False Discovery Rate FDR^67^, accepting an FDR of 0.05. The sign of fold change values for the effect of season is with reference to the wet season, i.e. genes with positive and negative fold change values being wet and dry season-biased, respectively.

### Gene ontology analyses

We performed gene ontology (GO) gene set enrichment (GSE) analyses in Babelomics v. 5^68^, using the FatiScan module. As input, we used lists of genes (and their fold change) that were significantly season-biased in thorax, abdomen or both body parts, and the Argot2 GO annotation file. The GSE analyses yield a log odds ratio for enrichment associated with each GO term, with the same sign as fold change values in expression: positive for GO terms enriched among wet season-biased genes and negative for GO terms enriched among dry season-biased genes. The associated *P-*values are corrected for multiple comparisons^67^, and we only report GO terms with FDR < 0.1 and that had more than three genes associated with it. To summarise and visualise long lists of GO terms we used REVIGO^69^ with the following parameters: default similarity (0.7), default semantic similarity measure (SimRel), *Drosophila melanogaster* database, including the log odd ratios from the GSE analyses (with higher absolute values is better). We visualised the output in scatterplots.

### Expression reaction norms and other downstream analyses

For downstream analyses, we normalised expression data in edgeR v. 3.10.0^66^ using trimmed methods of means (TMM), transformed to counts per million (CPM) and subsequently log_10_ transformed (for cluster analysis and PCA) or Z transformed (for reaction norm analyses and cross-environment genetic correlations). Cluster analyses were performed by constructing a neighbour joining tree from the Euclidian distance matrix computed from the normalised expression data using the R package “ape”^70^. PCA were calculated by single-value decomposition using the R function prcomp. To assess the association between Principal Components (PCs) and experimental factors, two-way ANOVAs were performed on the scores of each PC separately, with seasonal environment, family and their interaction as fixed factors. We computed family-specific reaction norms for each gene by fitting, for each family separately, the normalised expression data for that gene in a general linear model with season as the sole predictor. This yielded, for each family separately, the intercept and slope of this model, which was then used to calculate coefficient of variance across the seven full-sib families for both the intercept and slope. Differences in the transcriptome-wide distribution of these coefficients of variance were tested with two-sample Wilcoxon signed-rank tests (two-sided, using the function wilcoxsign-test from R package “coin”). We calculated cross-environment genetic correlations in normalised expression for each gene by averaging gene expression per family within each seasonal environment (*N* = 4–6 females per tissue per family per season) and calculating Pearson’s correlation coefficient using the expression of each of seven families in the wet season and in the dry season. Differences in log_2_ fold change values for the same genes but due to different experimental factors were tested with two-sided two-sample Wilcoxon signed-rank tests, testing whether log_2_ fold change values differed significantly from zero was done using one-sample Wilcoxon signed-rank tests, and testing whether absolute expression differed between gene repertoires were tested using two-sided Mann–Whitney *U*-tests. We generally preferred non-parametric tests as these are more robust to heteroscedasticity and differences in sample sizes. Log_2_ fold changes were calculated in edgeR, and those involving family (as main effect or in interaction with another factor) were calculated using all six mutually orthogonal contrasts between the seven families, and averaged across contrasts. Using maximum log_2_ fold change across contrasts rather than mean yielded similar results.

### Sequence polymorphism across the transcriptome

In order to assess footprints of selection in different gene repertoires, we quantified nucleotide diversity (*π*) and Tajima’s D for each transcript expressed in the abdomen. We used angsd^71^ to calculate pairwise nucleotide diversity *π* and Tajima’s D from the abdomen RNA-seq reads (aligned using Bowtie2 v. 2.2.3 to our Evigene-enriched reference transcriptome), restricting analyses to the coding sequence of each transcript. First, we used the angsd doSaf and realSFS commands to calculate genotype likelihoods and the folded site frequency spectrum from the bam files. Second, we used this site frequency spectrum as prior to calculate diversity measures (e.g. *π*, Tajima’s D), using the angsd commands doThetas followed by thetaStat make_bed and do_stat. This yielded diversity measures for 14,907 transcripts that were also expressed in the abdomen. Differences in Tajima’s D and *π* between different gene repertoires were tested using two-sided Mann–Whitney *U*-tests, implementing random permutations if sample sizes varied substantially between groups (with 1000 random draws of same sample size as the test group from all genes in the transcriptome).

### Comparisons with *Pieris rapae*

In order to identify *B. anynana* orthologs in *P*. *rapae*, we used blastx with *B. anynana* transcripts as a query against the *P*. *rapae* proteins as database, based on the recently sequenced *P*. *rapae* genome^72^, and retained only hits with bitscore >100 and evalue <10^−^^5^. Of the 15,049 *B. anynana* transcripts expressed in abdomen, 10,466 showed a significant hit to *P*. *rapae*. To estimate Tajima’s D and *π* in *P*. *rapae* coding sequence, we used whole genome Pool-Seq data from a single population (24 individuals). Briefly, adult *P*. *rapae* butterflies were collected during the summer of 2014, just south of Barcelona, Spain (Delta del Llobregat). DNA was extracted from thorax material using a modified version of a salt-extraction protocol. Library construction (PCR-free, paired end, 500 bp insert size), and sequencing (Illumina HiSeq 4000, 100 bp PE reads) was performed at BGI. Fastq data were quality-filtered to a minimum PHRED score of Q10, with ends trimmed of adapters and low-quality bases, and screened for common contaminants using bbduk2 (BBMap v35.69, http://sourceforge.net/projects/bbmap/). The Pool-seq data were mapped to the genome using Next-Gen Mapper v0.4.10 with an identity cutoff of 90% to minimise mapping bias^64^. SAMtools v1.2 was used to filter the mapped data for only correctly mapped paired-ends reads, after which a mpileup file was created for further analysis^63^. Using Popoolation v1.2.2, Tajima’s D and nucleotide diversity (*π*) were calculated per gene using the variance-at-position.pl script^73^ and a gtf annotation file. Annotation using the *B. anyana* evigene protein set was conducted with SPALN v2.1.2, an exon boundary-aware protein to genome alignment program^74^. The resulting gff file was converted to gtf using an in-house script.

All statistical analyses were performed in R v. 3.3.3^75^. Script is available at Figshare (doi: 10.6084/m9.figshare.4834031).

### Data availability

Raw RNA sequencing reads are available at NCBI Sequence Read Archive, BioProject ID PRJNA376691. The transcriptome assembly, expression raw count data, and R script are available at Figshare (doi: 10.6084/m9.figshare.4834031). Any other scripts are available from the authors upon request.

## Electronic supplementary material

Supplementary Information

Description of Additional Supplementary Files

Supplementary Data 1

Supplementary Data 2

Supplementary Data 3

Supplementary Data 4
