# Supplementary material for: Strong phenotypic plasticity limits potential for evolutionary responses to climate change
Source: Nat Commun. 2018 Mar 8;9:1005. doi: 10.1038/s41467-018-03384-9 (PMC5843647; doi:10.1038/s41467-018-03384-9)
Supplement: Supplementary file 1 — Supplementary Information [file 41467_2018_3384_MOESM1_ESM.pdf]

Supplementary Information for "Strong phenotypic plasticity limits potential for evolutionary responses to climate change"

Oostra et al.

Supplementary Notes

Seasonal plasticity across the transcriptome: systemic and tissue-specific components

Examining the seasonal plasticity programme in more detail revealed tissue-specific and systemic components, both at the level of individual genes and functional processes. A total of 2,115 genes showed the same response to the seasonal environment in both body parts, representing 14 and 17% of the abdomen and thorax transcriptome, respectively. This systemic plasticity programme is characterised by 89 enriched GO terms, which can be grouped into processes related to steroid hormone signalling, immunity, regulation of transcription (including DNA methylation), lipid metabolism, growth (including cell division DNA replication), protein turnover and oxidoreductase activity (Figure 2a; Supplementary Data 2; Supplementary Fig. 8a, b). We found particularly strong season bias among genes involved in Ecdysone and Juvenile hormone signalling or response, such as *JH binding proteins*, *JH acid methyltransferase*, *Ecdysone Receptor*, and *Urbain*. Other genes with strong season bias in both body parts included *Vitellogenin* and its receptor, Cuticular proteins, many lipases, and some immune-related genes including *Hdd1* and *Lebocin B* (Supplementary Data 1).

In addition to the systemic plasticity genes, 4,767 genes were season-biased in the abdomen but not the thorax (Figure 2c), representing 32% of the genes in the abdomen transcriptome. This number includes genes that were expressed in both body parts as well as genes absent from the thorax transcriptome. The abdomen-specific plasticity genes were enriched for 155 GO terms, which could roughly be grouped into processes related to development, mannose metabolism, and response to oxidative stress as well processes also enriched among the systemic genes (Supplementary Data 2; Supplementary Fig. 8c, d). Genes showing a strong season bias exclusively in this tissue included *Vitellin-degrading protease* and *Vitelline membrane associated protein P30*, *Serine-type endopeptidase*, *Catalase*, *Heat Shock Protein 20.1*, and *Trehalase*. (Supplementary Data 1).

Similarly, 3,752 genes were differentially expressed between the seasons only in the thorax, representing 30% of the thorax transcriptome. They showed significant enrichment for 186 GO terms, which roughly grouped into processes related to mitochondrial protein translation and to the actin cytoskeleton, as well as processes also enriched among the systemic genes (Figure 2d; Supplementary Data 2; Supplementary Fig. 8e, f). Among the most strongly season-biased genes in thorax were *Attacin*, *19.5 kDa Heat Shock Protein*, and *DNA cytosine-5 methyltransferase* (Supplementary Data 1).

Some of the overrepresented GO terms for seasonal plasticity identified separately in abdomen or thorax are shared, reflecting an additional systemic signature of adaptive plasticity that is only apparent at the level of functional processes, not individual genes. In particular, we identified 37 such shared GO terms showing the same pattern of seasonal bias between the thorax and abdomen (Supplementary Data 2). Together, these processes represent an additional 865 unique genes in the systemic plasticity programme that at the individual gene level appeared restricted in their plasticity response to either abdomen (398 genes) or thorax (467). Thus, while individual genes may be involved in plasticity in one body part and not the other, they may still contribute to functional processes that are shared between the body parts, and these genes can therefore be considered part of the systemic plasticity programme.

However, the large majority of GO terms identified in each body part separately is uniquely enriched in that body part and not the other (109 and 140 for abdomen and thorax, respectively).

Interestingly, we also identified 781 individual genes that showed opposite patterns of season bias between the two body parts, i.e. their expression was wet season-biased in one body part and dry season-biased in the other body part, representing a substantial fraction (6%) of the shared transcriptome. These genes were enriched for 37 GO terms (Figure 2b; Supplementary Data 2). In addition, the abdomen- and thorax-specific plasticity genes were enriched for 41 GO terms that overlapped but showed opposite patterns of seasonal response across the body parts. These processes are mostly related to ubiquitination, cell division, lipid metabolism, chitin metabolism, and translation, likely reflicting trade-offs between the body parts that differ across the seasons, for example in investment in growth, storage and turnover of resources. Genes in this group included *Bombyrin*, *Larval cuticle protein 16/17*, *Neuropeptide Y* and several chitin-related proteins (Supplementary Data 1).

Taken together, we identify a broad, genome-wide transcriptional programme involved in seasonal plasticity. While a substantial part of the transcriptional response is systemic, reflecting an integrated and coordinated environmental response across the body, the largest component to the seasonal transcriptional response is tissue-specific, reflecting modular and independent responses to the seasonal environment. See Supplementary Data 1 for a full list of season-biased genes.

Reduced genetic variation for plasticity

Expression of 1% of genes (160 and 146 genes in abdomens and thoraces, respectively, and 20 in both, was significantly affected (FDR < 0.05) by the interaction between seasonal environment and family, i.e. genotype-by-environment interaction (GxE; Figure 3a, b). This limited set of genes included genes coding for *Zinc finger proteins*, *Gloverin*, *Triacylglycerol lipase*, *Alcohol dehydrogenase*, *Reverse transcriptase*, *Cytochrome P-450*, *Disco-related protein*, *Heat shock protein 60*, and *Serine/threonine-protein kinase rio3* (Supplementary Data 3). It was not enriched for any GO terms, with the exception of "extracellular vesicular exosome" (GO:0070062) in the abdomen (5 genes, adjusted p = 0.028).

Tajima's D and pairwise nucleotide diversity in coding sequence

In order to test the hypothesis that the observed lack of inter-family variation in plasticity is due to past positive or purifying selection on reaction norms, we quantified Tajima's D, the difference between the fraction of pairwise nucleotide differences and segregating sites[1], for each expressed gene and compared it across gene repertoires. Genes differing in expression plasticity across families (i.e. gene-by-environment interaction or GxE) showed a reduction from 0.40 to 0.32 in median Tajima's D compared to genes showing no such GxE (Mann Whitney test, p = 0.041, in top 5% extreme p values of 1000 randomly drawn genesets of same sample size). This decreased Tajima's D was not observed for other gene repertoires, consistent with purifying selection for reaction norms (Supplementary Fig. 11c). Despite the reduction, Tajima's D in this geneset was significantly higher than zero (one-sample one-sided Wilcoxon signed rank test $p < 10^{-15}$).

Pairwise nucleotide diversity (π) was 6% elevated in genes showing significant inter-family expression variation, compared to genes without family effects (Mann-Whitney U test $p < 10^{-13}$; Supplementary Fig. 12b). This is consistent with heritable gene expression variation being at least partly driven by nucleotide variation within coding sequence, possibly due to linkage with cis-regulatory regions elsewhere in the gene. Average π in coding sequence across all genes in the transcriptome was 0.0068, and the fraction of segregating sites (Watterson's theta) was 0.0061.

Finally, Tajima's D was slightly but significantly higher in season-biased genes compared to those not showing an effect of the seasonal environment (Supplementary Fig. 11a), which may be indicative of increased balancing selection in the form of antagonistic selective pressures across the seasons, favouring alternative alleles in each season. At the same time, average π was also slightly (3%) increased (Supplementary Fig. S12a), which may instead point to relaxed selection in season-biased genes.

Developmental food stress

Adults that were food-deprived for a limited period of larval development show significant differential expression in only 25 genes in abdomen and none in thorax (Supplementary Fig. 1). Since the effect of developmental stress may be different in different seasons, we additionally tested the effect of food stress within each season separately. This revealed four stress-induced genes in the thorax, but only in the dry season, none in the wet season. In contrast, in the abdomen there were 19 genes affected by food stress, most of which in the wet season (Supplementary Table 2).

Assessing the effect of food stress separately for specific gene repertoires revealed subtle stress-induced shifts in seasonal expression patterns, slightly decreasing transcriptional divergence between the seasons. In the abdomen, where the reproductive tissues are located, the typical dry season expression patterns became slightly less distinct under stress. Dry season genes, normally higher expressed in the dry season compared to wet (FDR < 0.05, fold change > 2), showed a stress-induced reduction in abdominal expression in the dry season, and wet season genes showed a stress-induced up-regulation in the dry season (Supplementary Fig. 14a, left panel; one-sample Wilcoxon signed rank test $p < 0.0005$). Thus, dry season butterflies under stress become more wet-season like in their abdominal transcriptional profile, indicating a stress-induced emergency response comparable to a terminal reproductive investment (*cf.* [2]). In contrast, this response was absent for wet season butterflies (Supplementary Fig. 14a, right panel), which presumably are already physiologically set up to reproduce maximally. We observe a similar down-regulation of dry season genes upon stress ($p < 0.0005$) but unlike in the abdomen there is no up-regulation of wet season genes, consistent with reproductive functions being restricted to the abdomen. The down-regulation of dry season genes in the thorax was also observed under wet season conditions, further decreasing their already low expression, although this response was not very pronounced ($p < 0.05$; Supplementary Fig. 14b). Thus, stress pushed the typical dry season morph towards a slightly more wet season-like transcriptional profile, partly driven by an emergency response in the abdomen comparable to a terminal reproductive investment.

Robustness of results to various mapping and filtering strategies

In order to ensure that our result were not biased by a particular choice of filtering, mapping, transcript abundance estimation, or expression filtering strategy, we repeated differential expression analyses a) without the low expression filter, and b) using alternative combinations of transcriptome filtering, mapping programs and abudance estimation approaches (detailed in Supplementary Table 5), in addition to the main approach described in the Materials & Methods. Although the total numbers of expressed genes varied over an order of magnitude between the most restrictive and most permissive approaches, the relative proportions of genes showing a significant (FDR < 0.05) effect of seasonal environment, food stress, genetic background, and any two- and three-way interaction were qualitatively similar. In all cases, we observed the same lack of genes whose expression was affected by the interaction between seasonal environment and family (i.e. GxE), compared to the large number of gense whose average expression differed significantly between families. This was the case for the analyses without expression filtering (Supplementary Fig. 3) and for the analyses using different combinations of mapping approaches (Supplementary Fig. 2, Supplementary Table 1).

We also performed a more restrictive differential expression analysis, calling genes differently expressed only if, in addition to differing significantly with FDR < 0.05 also showed absolute fold change larger than two. Although the total numbers of these differently expressed large-effect genes were drastically lower than without this additional threshold, the relative proportion of genes showing an effect of the seasonal environment, food stress, genetic background, and any two- and three-way interaction was qualitatively similar. In particular, we observed a similarly low number of genes with significant gene-by-environment effects on expresssion compared to the many genes showing significant inter-family differences in average expression (Supplementary Fig. 4).

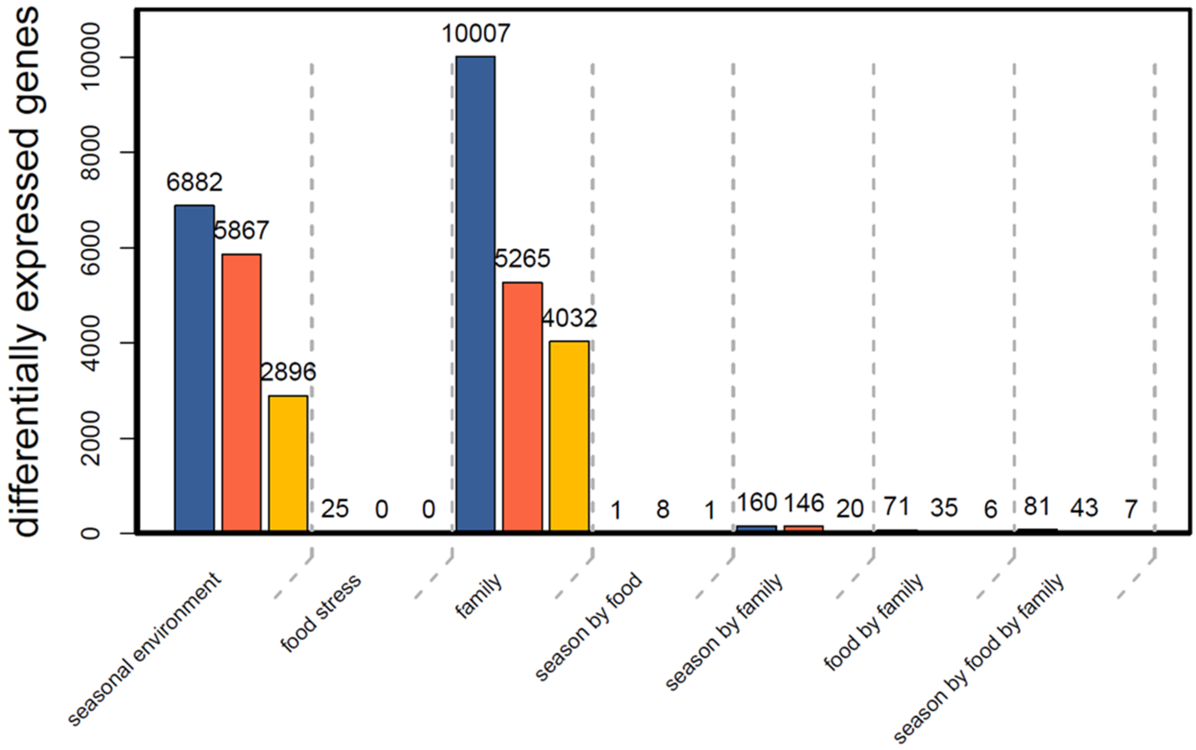

Supplementary Figure 1. Pervasive seasonal plasticity and intra-population genetic variation but low genetic variation for plasticity across the transcriptome. Differential expression analyses identify thousands of genes significantly affected by seasonal environment and genetic background, and relatively few genes affected by the gene-by-environment interaction. The vertical axis indicates numbers of significantly differentially expressed genes (FDR < 0.05) due to seasonal environment, food stress treatment, genetic background, or their interactions in edgeR general linear models, with genes affected in abdomen, thorax, and in both tissues indicated with blue, red, and orange bars, respectively. Numbers above each bar indicate the number of differentially expressed genes for that particular factor and body part. A total of 15,049 genes were expressed in abdomen, 12,567 in thorax, and 12,309 in both body parts.

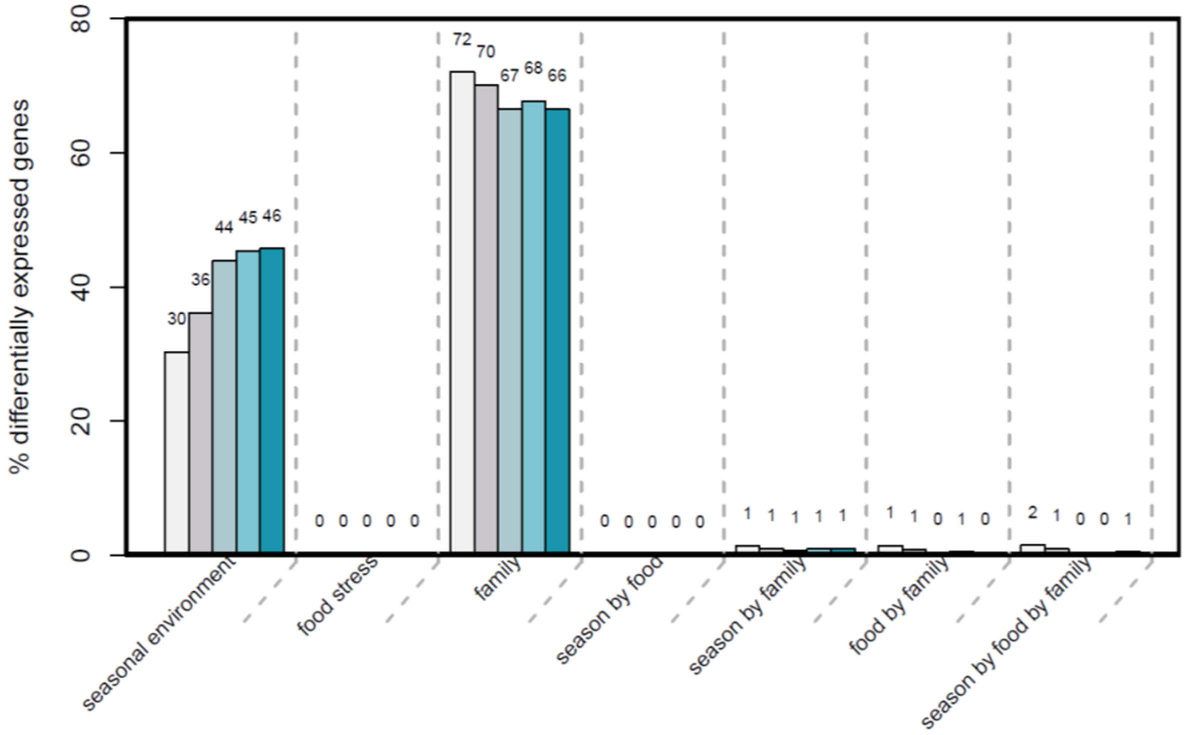

Supplementary Figure 2. Robustness of differential expression analyses to alternative mapping approaches. Differential expression analyses of the full-factorial experimental design were performed on expression data produced using four alternative mapping approaches (in addition to the main method). The vertical axis indicates numbers of significantly differentially expressed genes (FDR < 0.05) due to seasonal environment, food stress treatment, genetic background, or their interactions in edgeR general linear models, with genes affected in each of the five approaches (described in Supplementary Table 5) indicated with different colours. Numbers above each bar indicate the percentage of differentially expressed genes for that particular factor and mapping approach. See Supplementary Table 1 for absolute numbers.

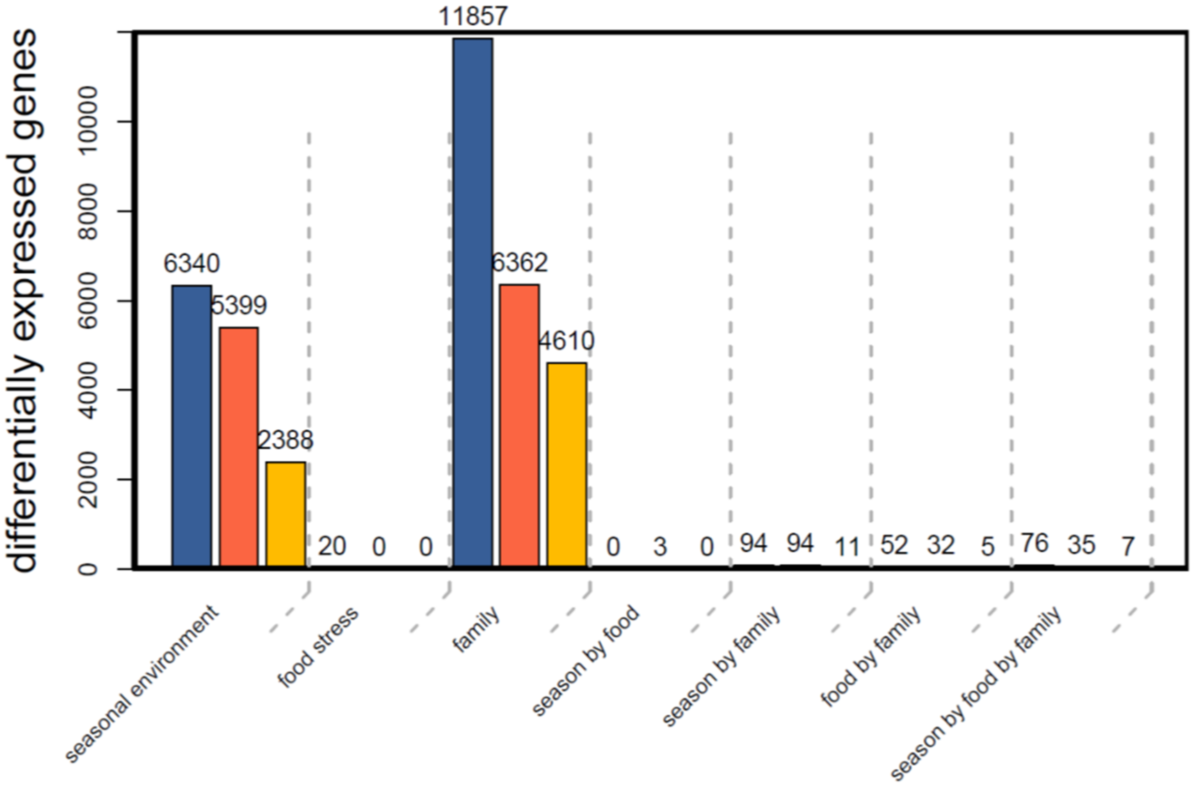

Supplementary Figure 3. Robustness of differential expression analyses to low expression filtering. Differential expression analysis on expression data that were not filtered for low expression, i.e. retaining all genes that were expressed in at least one individual. Compare with the main analysis, where genes were removed that were expressed in less than 3 samples as well as genes with average expression < 0.25 CPM (see Methods). The vertical axis indicates numbers of significantly differentially expressed genes (FDR < 0.05) due to seasonal environment, food stress treatment, genetic background, or their interactions in edgeR general linear models, with genes affected in abdomen, thorax, and in both tissues indicated with blue, red, and orange bars, respectively. Numbers above each bar indicate the number of differentially expressed genes for that particular factor and body part. A total of 34,970 genes were expressed in abdomen, 30,734 in thorax, and 29,957 in both body parts.

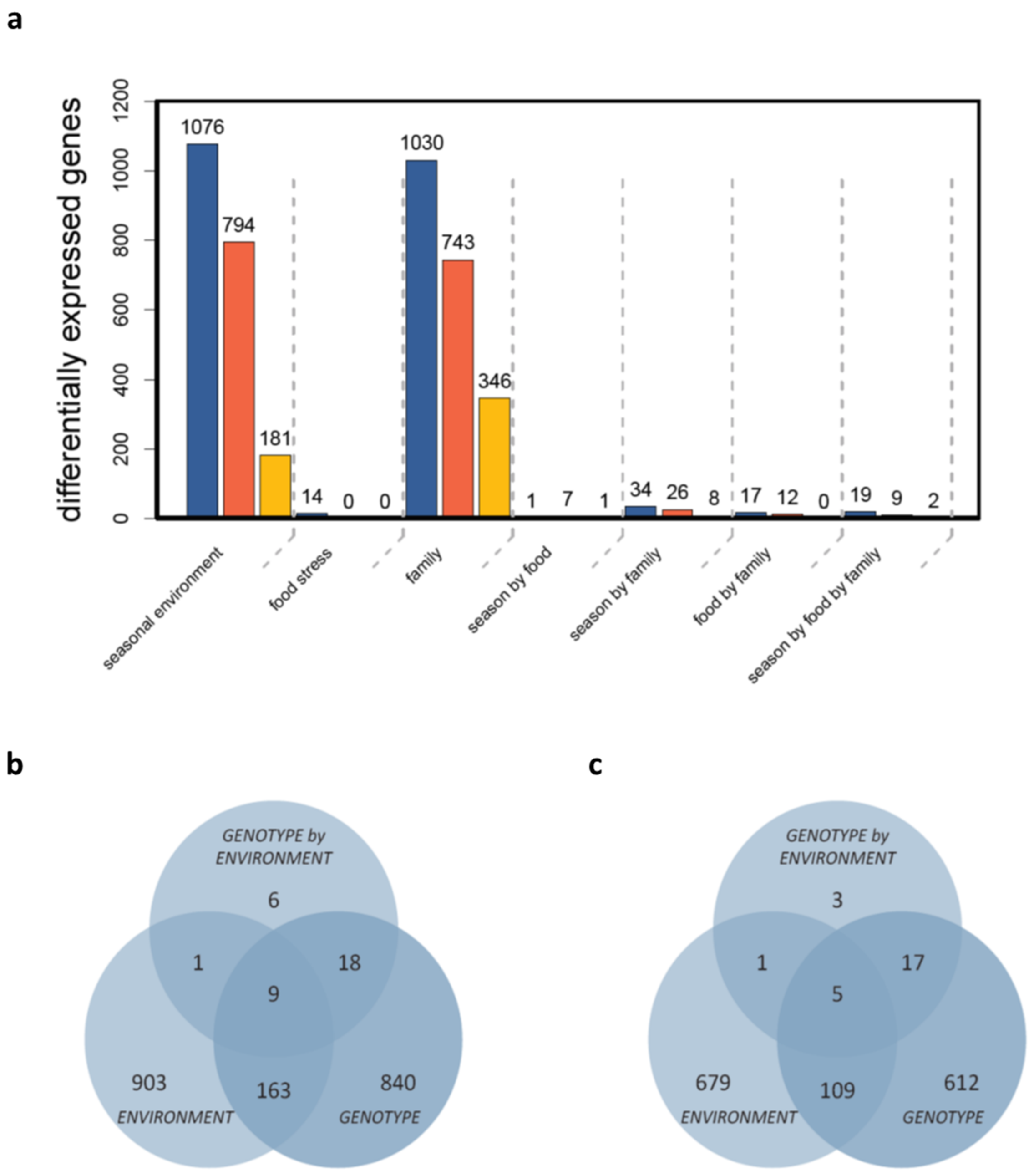

Supplementary Figure 4. Robustness of differential expression analyses to fold change threshold. A more restrictive differential expression analysis was performed, calling genes differently expressed only if, in addition to differing significantly with FDR < 0.05 also showed absolute fold change larger than two. **a)** Summary of differential expression analysis. The vertical axis indicates numbers of significantly differentially expressed genes (FDR < 0.05 and absolute fold change > 2) due to seasonal environment, food stress treatment, genetic background, or their interactions in edgeR general linear models, with genes affected in abdomen, thorax, and in both tissues indicated with blue, red, and orange bars,

respectively. Numbers above each bar indicate the number of differentially expressed genes for that particular factor and body part.

b) and **c)** An order of magnitude more genes show significant differential expression due to seasonal environment and genetic background than due to the interaction between environment and genetic background for abdomen **(b)** and thorax **(c)**. Within each Venn diagram, numbers of differentially expressed genes (FDR < 0.05 and absolute fold change > 2) are indicated for seasonal environment (left), genetic background (right), and their interaction (top), as well as overlap in responses among genes.

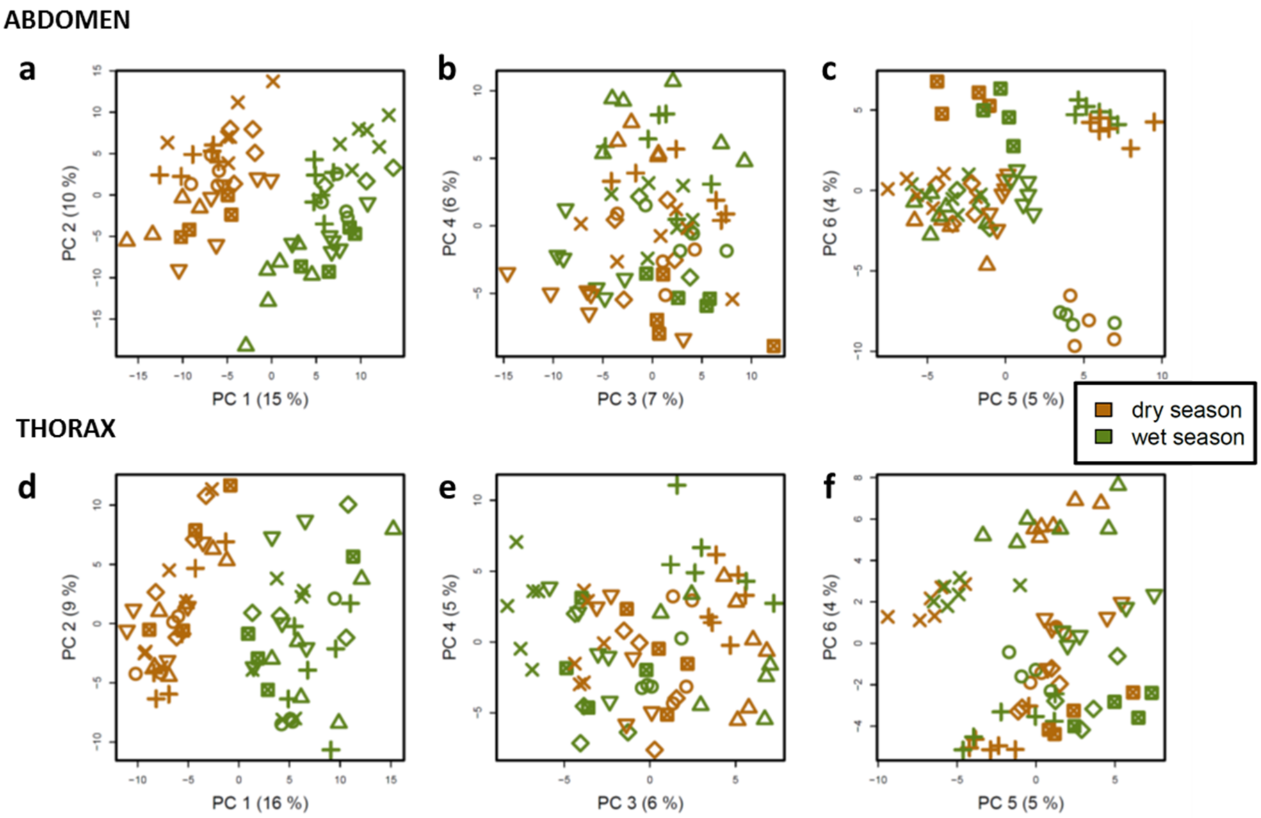

Supplementary Figure 5: Principal Components Analysis (PCA) reveals seasonal environment and genetic background as major drivers of whole-transcriptome expression profiles for abdomen (a-c) and thorax (d-f). Individuals are plotted in Principal Component (PC) space for PC 1 through 6, with percentage variance explained by each PC indicated on the axes. Individuals reared in wet and dry season environments are represented in green and brown, respectively, and individuals from different full-sib families have different symbols.

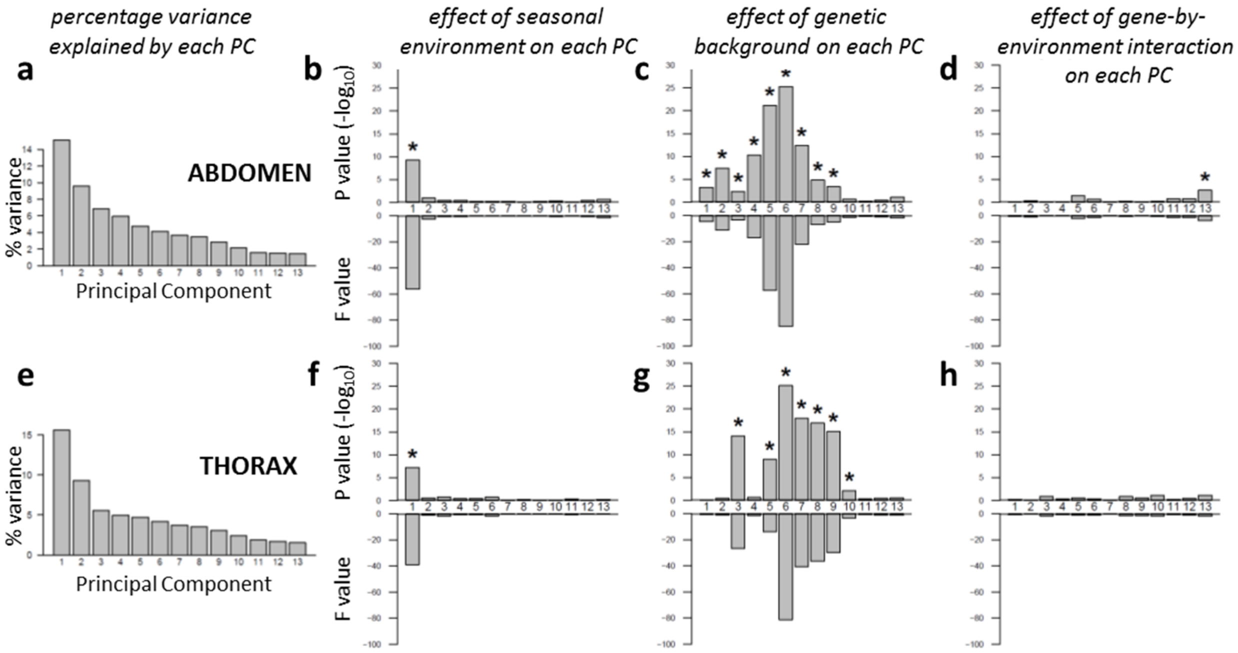

Supplementary Figure 6. Significant associations of whole-transcriptome PCs with seasonal environment and genetic background but not genetic variation for plasticity. (a, e) Percentage transcriptional variance explained by each of first 13 PCs is plotted for abdomen **(a)** and thorax **(e)**. **(b-d, f-h)** The seasonal environment and genetic background, but not their interaction, associate significantly with major PCs. P values (upper barplots) and F statistics (lower barplots) are shown on the vertical axes for two-way ANOVAs with seasonal environment (left panels **b, f**), genetic background (middle panels **c, g**) and their interaction (right panels **d, h**) as fixed effects and PC 1 through 13 as dependent variables (plotted along each horizontal axis), for abdomen (upper panels **b-d**) and thorax (lower panels **f-h**). Asterisks indicate a significant association (FDR < 0.05). In thorax, none of the first 13 Principal Components (together accounting for 62% of total variance) associated with the interaction between seasonal environment and genetic background (FDR > 0.49, F < 2.0), while in abdomen only PC 13 (accounting for 1.5% of total variance) was significantly affected by the interaction between seasonal environment and genetic background (FDR = 0.03, F = 3.9). In contrast, major PCs accounting for 15 to 56% of total variance are significantly (FDR < 0.05) associated with the seasonal environment or the genetic background.

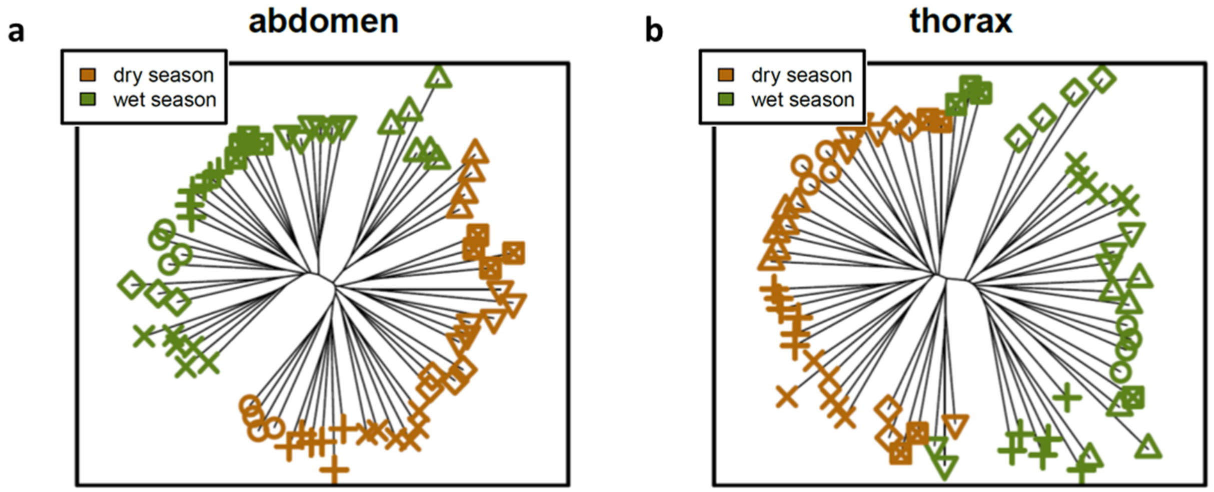

Supplementary Figure 7. Clustering of gene expression by seasonal environment and full-sib family. (a, b). Neighbour joining trees from Euclidian distances of whole-transcriptome expression profiles for abdomen **(a)** and thorax **(b)** separate individuals reared in wet (green) or dry (brown) season conditions, as well as individuals from different full-sib family (different symbols).

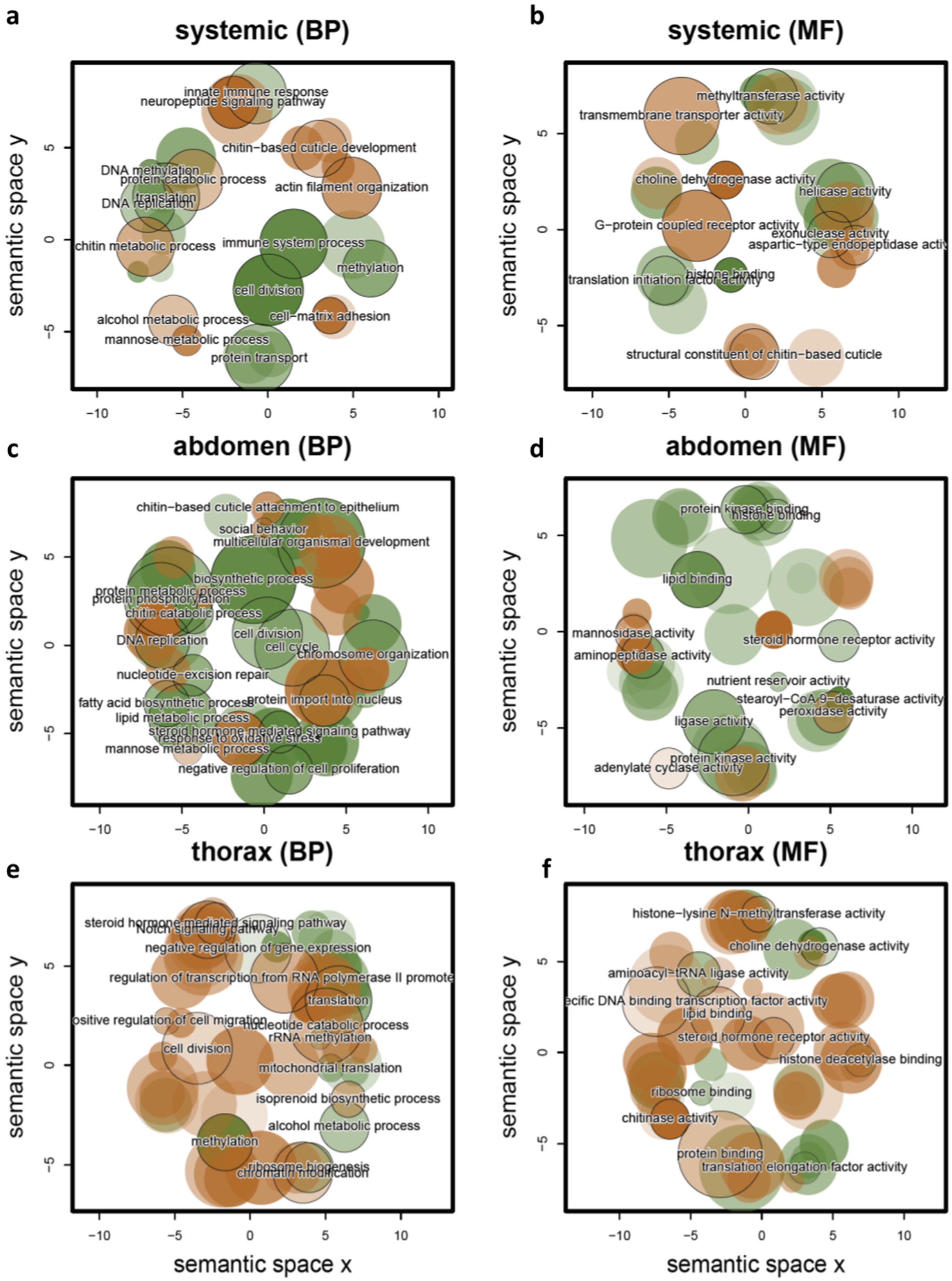
a
systemic (BP)
b
systemic (MF)
c
abdomen (BP)
d
abdomen (MF)
e
thorax (BP)
f
thorax (MF)
semantic space x
semantic space y
innate immune response
neuropeptide signaling pathway
chitin−based cuticle development
DNA methylation
protein catabolic process
actin filament organization
translation
DNA replication
chitin metabolic process
immune system process
methylation
cell division
alcohol metabolic process
cell−matrix adhesion
mannose metabolic process
protein transport
methyltransferase activity
transmembrane transporter activity
choline dehydrogenase activity
helicase activity
G−protein coupled receptor activity
exonuclease activity
translation initiation factor activity
histone binding
structural constituent of chitin−based cuticle
chitin−based cuticle attachment to epithelium
social behavior
multicellular organismal development
biosynthetic process
protein metabolic process
protein phosphorylation
chitin catabolic process
DNA replication
cell division
cell cycle
chromosome organization
nucleotide−excision repair
fatty acid biosynthetic process
protein import into nucleus
lipid metabolic process
steroid hormone mediated signaling pathway
response to oxidative stress
mannose metabolic process
negative regulation of cell proliferation
protein kinase binding
histone binding
lipid binding
mannosidase activity
steroid hormone receptor activity
aminopeptidase activity
nutrient reservoir activity
stearoyl−CoA 9−desaturase activity
peroxidase activity
ligase activity
protein kinase activity
adenylate cyclase activity
steroid hormone mediated signaling pathway
Notch signaling pathway
negative regulation of gene expression
translation
nucleotide catabolic process
rRNA methylation
cell division
mitochondrial translation
isoprenoid biosynthetic process
alcohol metabolic process
methylation
ribosome biogenesis
chromatin modification
histone−lysine N−methyltransferase activity
choline dehydrogenase activity
aminoacyl−tRNA ligase activity
lipid binding
steroid hormone receptor activity
histone deacetylase binding
ribosome binding
chitinase activity
protein binding
translation elongation factor activity

Supplementary Figure 8: Gene Set Enrichment (GSE) analysis of systemic and tissue-specific components of the seasonal plasticity programme. Gene Ontology (GO) terms enriched among genes differentially expressed between dry and wet season are plotted in semantic space, with more similar terms grouped closer together. Wet and dry season-biased GO terms are plotted in green and brown, respectively, with opacity proportional to the extent of enrichment. Analyses for systemic (shared), abdomen-specific, and thorax-specific plasticity genes are displayed in top **(a, b)**, middle **(c, d)** and bottom rows **(e, f)**, respectively. “Biological Process” (BP) and “Molecular Function” (MF) GO terms are in left **(a, c, e)** and right **(b, d, f)** panels, respectively, and names of selected GO terms are labelled. See Supplementary Data 2 for a full list of all GO terms.

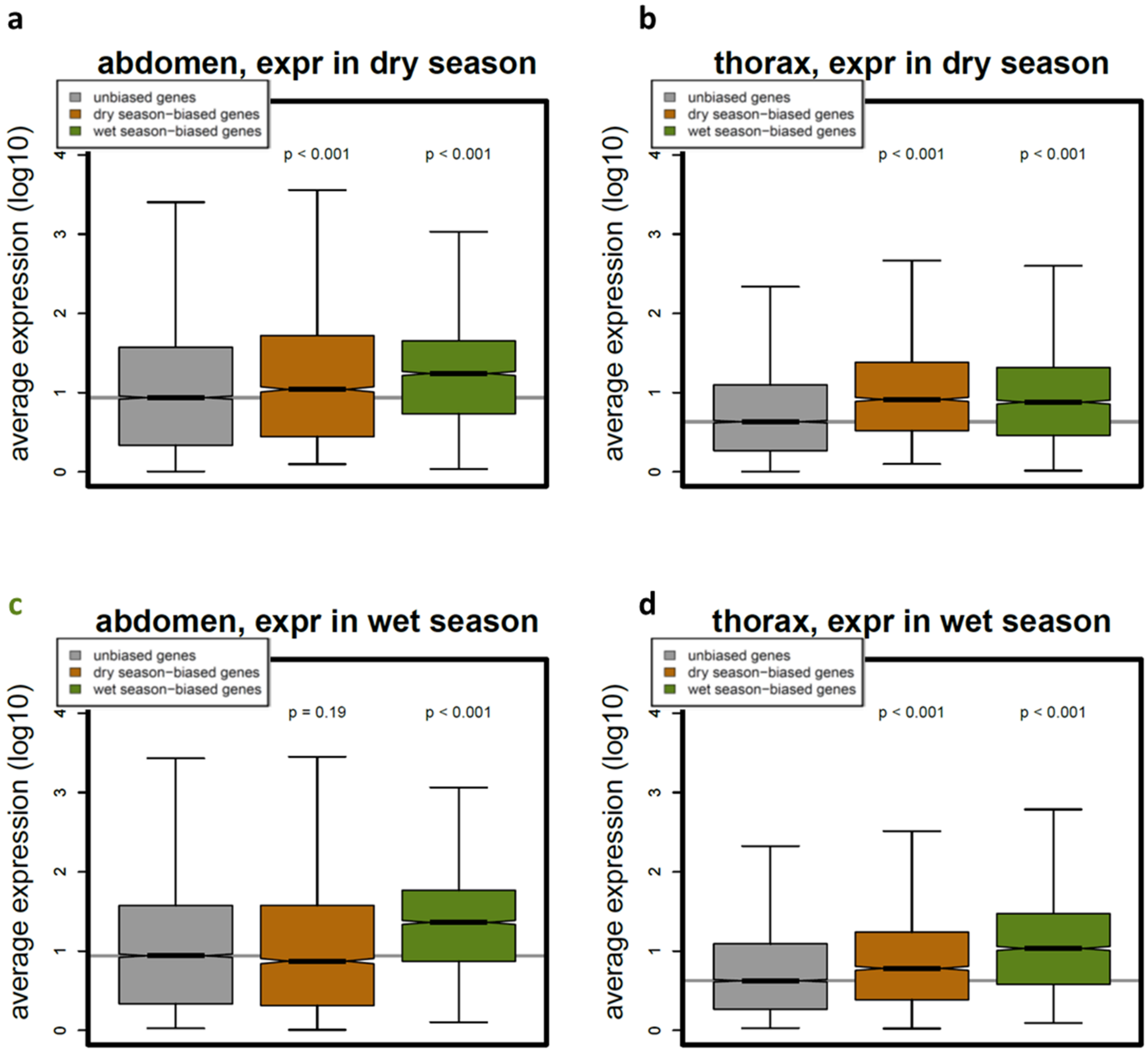

Supplementary Figure 9. Season-biased genes are more highly expressed than unbiased genes. In dry season conditions **(a, b; top row)**, average gene expression (log_{10} CPM) is significantly higher for season-biased genes (in brown and green for dry and wet season genes) compared to unbiased genes (in grey), while in wet season conditions **(c, d; bottom row)** average expression for wet season genes (in green) is significantly higher than for unbiased genes (in grey). Thus, for both abdomen **(a, c; left)** and thorax **(b, d; right)**, season-biased genes have higher expression than unbiased genes, as expected for plasticity genes involved in the seasonal phenotypes. P values above boxplots of season-biased genes indicate whether expression for genes in that group differs from expression of unbiased genes (two-sided Mann Whitney U tests). Upper whiskers are at the upper quartile plus 1.5x the interquartile range or at the maximum value (whichever is lowest), whereas lower whiskers extend to the lower quartile minus 1.5x the interquartile range or to the minimum value (whichever is highest).

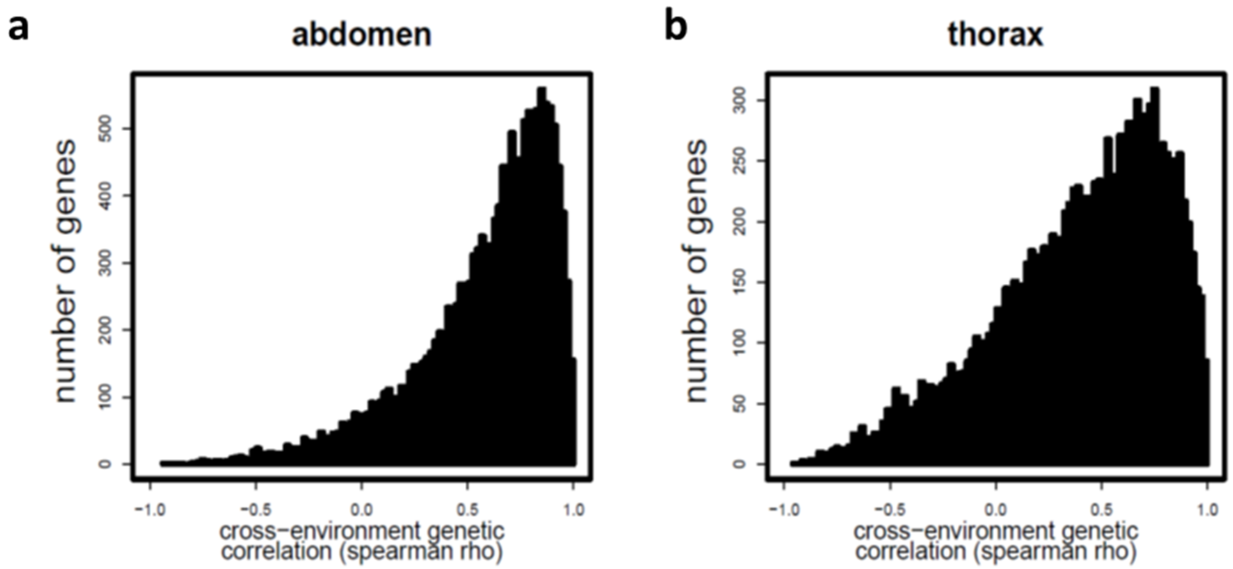

Supplementary Figure 10. Positive, high across-environmental genetic correlations in expression. The histograms show the distributions across all genes of Pearson's correlation coefficients for the correlation between average per-family expression across the two seasonal environments, for abdomen **(a)** and thorax **(b)**. This indicates that for many genes, expression in one season is genetically coupled with expression in the other season, and there is limited genetic variation for expression that is independent between the seasons.

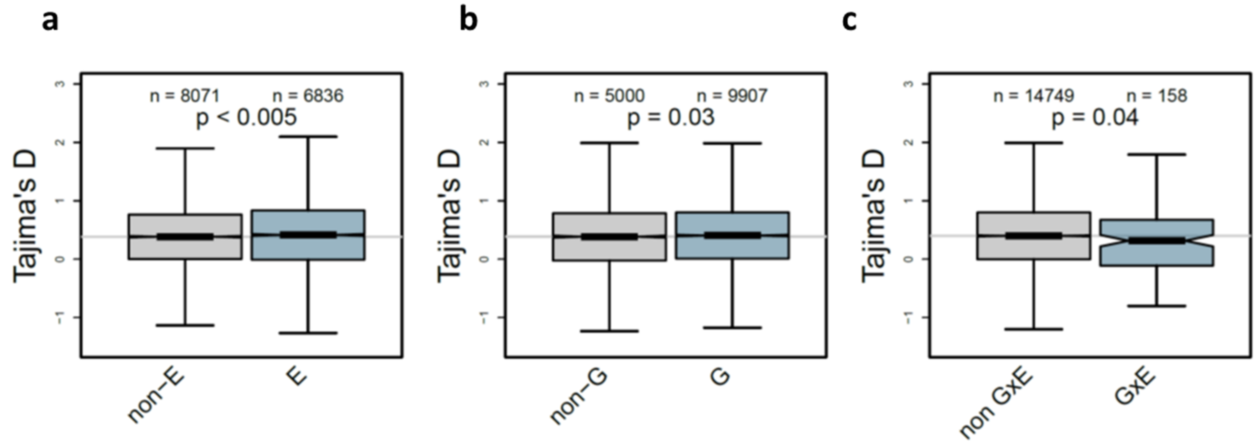

Supplementary Figure S11. Tajima’s D is reduced among genes showing gene-by-environment interaction in expression, consistent with purifying selection on reaction norms. Tajima’s D, a measure of DNA sequence polymorphism, is plotted for different gene repertoires, categorised based on their expression patterns, with p values from two-sided Mann Whitney U tests. **a)** Genes affected by the seasonal environment (season-biased genes (E) show elevated Tajima’s D compared to unbiased genes (non-E), suggesting increased balancing or relaxed selection in these genes. **b)** Genes showing significant expression variation across families (G) show elevated levels of Tajima’s D compared to genes not affected by genetic backgground (non-G). **c)** Season-by-family genes show reduced Tajima’s D compared to genes not showing GxE (non-GxE; unbiased genes (Mann Whitney two-sided $p = 0.041$), indicating an excess of rare alleles. This p value fell into the top 5% extreme p values when testing 1,000 randomly drawn gene sets of the same sample size from the whole transcriptome. Despite the reduction, Tajima’s D in this geneset was significantly higher than zero (one-sample one-sided Wilcoxon signed rank test $p < 10^{-15}$). Upper whiskers are at the upper quartile plus 1.5x the interquartile range or at the maximum value (whichever is lowest), whereas lower whiskers extend to the lower quartile minus 1.5x the interquartile range or to the minimum value (whichever is highest).

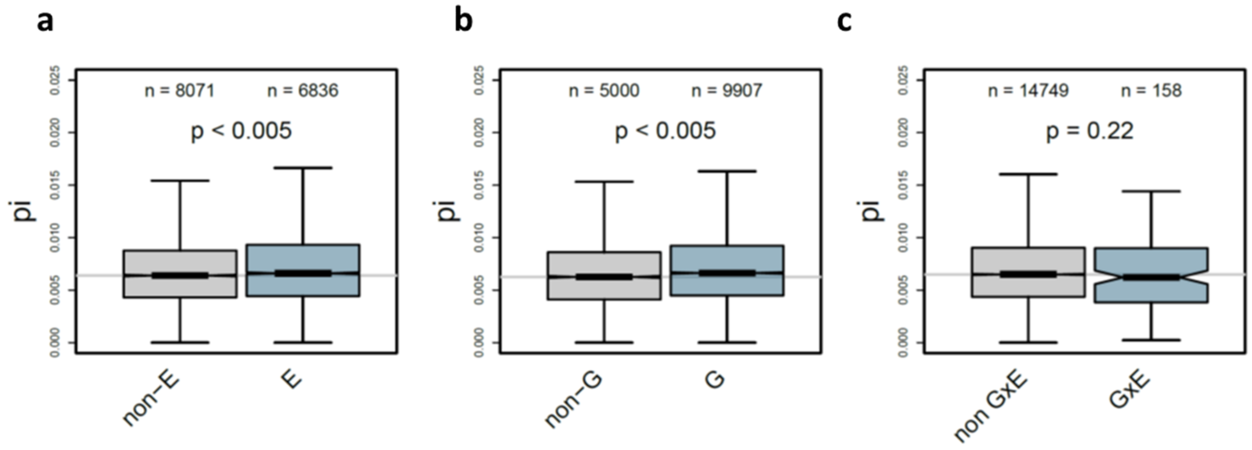

Supplementary Figure 12. Season-biased genes and genes showing inter-family variation in expression have elevated nucleotide diversity in coding sequence. Pairwise nucleotide diversity (π) is plotted for different gene repertoires, categorised based on their expression patterns. **a)** Genes affected by the seasonal environment (season-biased genes; E) show elevated nucleotide diversity compared to unbiased genes (non-E), suggesting increased balancing or relaxed selection in these genes. **b)** Genes showing significant expression variation across families (G) show 6% elevated nucleotide diversity compared to genes not affected by genetic background (non-G). This is consistent with heritable gene expression variation being at least partly driven by nucleotide variation within coding sequence, possibly due to linkage with cis-regulatory regions elsewhere in the gene. **c)** Genes showing a significant effect of gene-by-environment interaction on expression (GxE) show similar levels of nucleotide diversity compared to genes without a GxE effect (non-GxE). Sample sizes (numbers of genes in each category) are indicated above the boxplots, as are p values from two-sided Mann Whitney U tests. Upper whiskers are at the upper quartile plus 1.5x the interquartile range or at the maximum value (whichever is lowest), whereas lower whiskers extend to the lower quartile minus 1.5x the interquartile range or to the minimum value (whichever is highest).

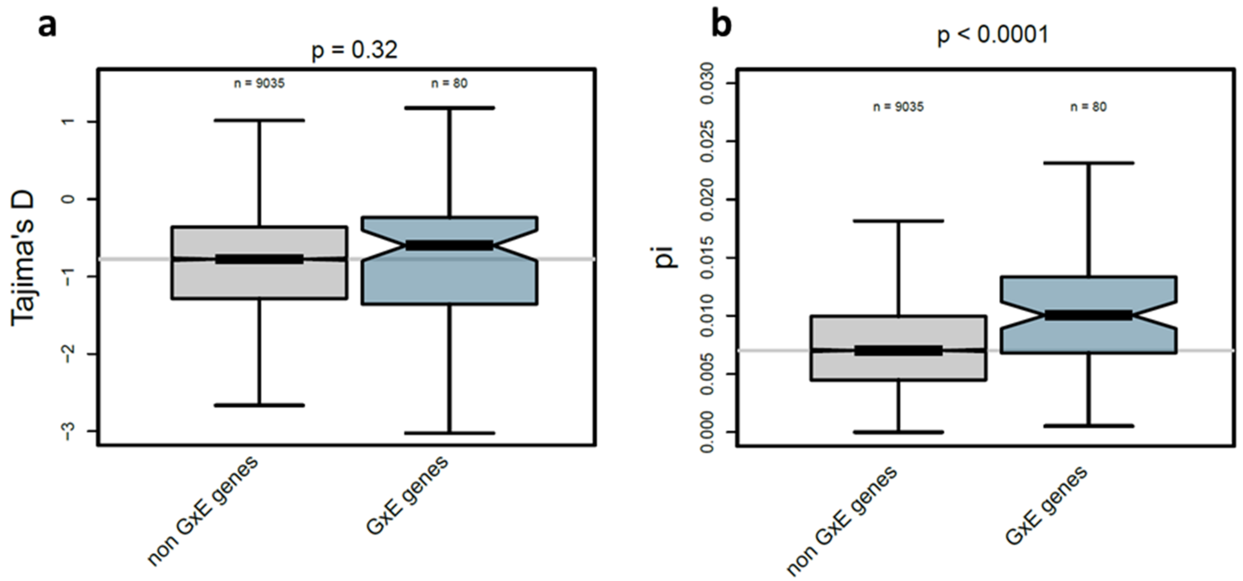

Supplementary Figure 13. Coding sequence polymorphism and diversity in *Pieris napi* for genes showing gene-by-environment interaction in *B. anynana*. a) *P. napi* orthologs of genes showing significant GxE in *B. anynana* show similar levels of polymorphism (Tajima's D) compared to genes not showing GxE. **b)** *Pieris napi* orthologs of genes showing significant GxE in *B. anynana* show increased levels of pairwise nucleotide diversity (π) compared to genes not showing GxE. P values above boxplots are for two-sided Mann Whitney U tests. Upper whiskers are at the upper quartile plus 1.5x the interquartile range or at the maximum value (whichever is lowest), whereas lower whiskers extend to the lower quartile minus 1.5x the interquartile range or to the minimum value (whichever is highest).

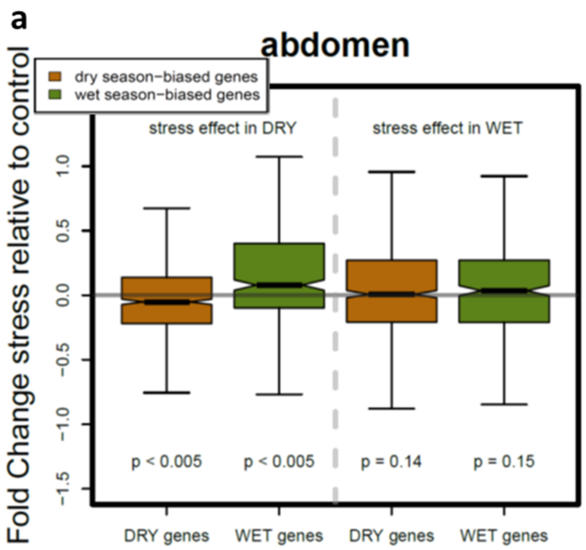

Supplementary Figure 14. Developmental food stress in the dry season induces a subtle reduction in seasonal transcriptional divergence. In the abdomen **(a)**, dry season genes (brown; normally having high expression in dry season and low expression in wet season) show downregulation upon stress in the dry season (left panel), but not in wet season (right panel). Wet season genes (green; normally having high expression in wet season and low expression in dry season) show upregulation in dry season (left panel), but not in wet season (right panel). Thus, in the dry season (left) the plasticity programme shifts towards wet season-like expression upon stress, while in the wet season this effect is absent. In the thorax **(b)**, a similar pattern is observed for dry season genes, which are downregulated upon stress in the dry season, but not for wet season genes, which are not upregulated upon stress in the dry season. Dry season and wet season genes were defined as genes differentially expressed between the seasonal environments (FDR < 0.05, Fold Change > 2). P-values below each boxplot indicate whether $\log_2$ Fold Change for each group of genes for the effect of food stress within each seasonal environment differs from zero (two-sided one-sample Wilcoxon signed rank tests). Upper whiskers are at the upper quartile plus 1.5x the interquartile range or at the maximum value (whichever is lowest), whereas lower whiskers extend to the lower quartile minus 1.5x the interquartile range or to the minimum value (whichever is highest).

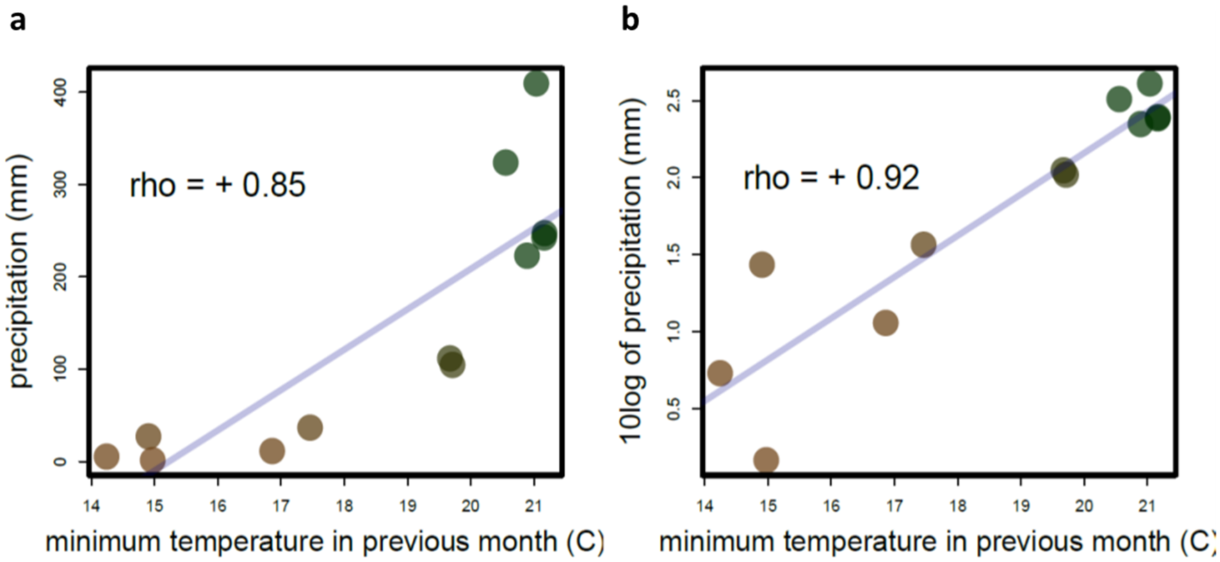

Supplementary Figure 15. Temperature as a reliable cue for seasonal progression in *B. anynana*'s natural habitat makes seasonal transitions highly predictable.

Monthly-averaged precipitation **(a)** and $\log_{10}$ of monthly-averaged precipitation **(b)** are highly correlated with monthly-averaged minimum temperature in the previous month ($\rho_{pearson}$ = + 0.85 and + 0.92) in Nkhata Bay in Malawi, where the laboratory population originated. Each dot represents a month, with shades from brown to green representing dry to wet season months. Climate data is for 1901-2009[3], downloaded 12 Oct 2016 via http://www.globalspecies.org/weather_stations/climate/429/157.

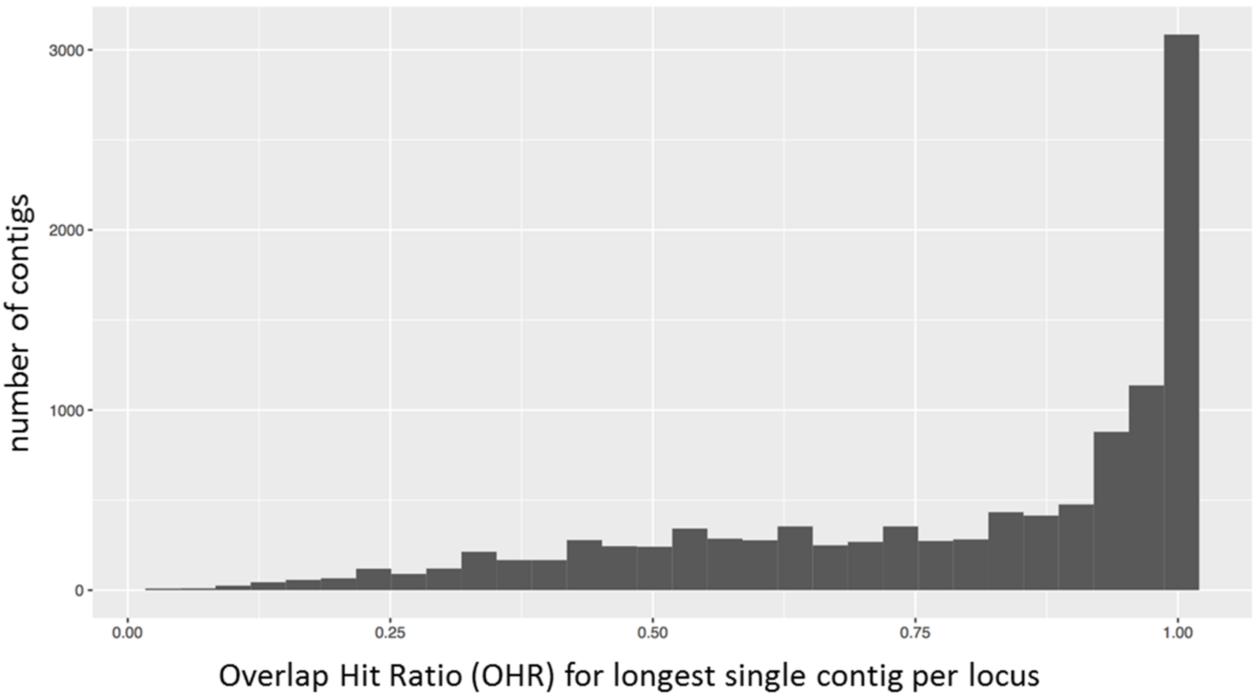

Supplementary Figure 16. High completeness of assembled contigs in *de novo* transcriptome assembly. The histogram shows the distribution of the Overlap Hit Ratio (OHR) for the longest single contig per locus in the transcriptome assembly. OHR was determined via blast of each protein sequence in the *Heliconius melpomene* genome against our *de novo* assembly (collapsed using Evigene; see Methods), and a best hit contig was identified. For each best hit relationship, OHR was estimated by dividing the length of the alignment by the length of the, effectively dividing the length of assembled contig by their expected length, with values near 1 indicating a nearly full length assembly assuming homology. For situations where a single protein hit mulitiple contigs, only the longest OHR was reported for that protein. See also Supplementary Table 4.

SUPPLEMENTARY TABLES

Supplementary Table 1. Results of differential expression analyses using alternative mapping approaches

Factor	approach 1	approach 2	approach 3	approach 4	main method
seasonal environment	15565	12644	5959	7289	6882
food stress	119	56	15	16	25
family	37012	24528	9028	10881	10007
season by stress	73	30	0	0	1
season by family	715	354	105	153	160
stress by family	753	317	58	81	71
season by stress by family	777	345	55	66	81
total expressed genes	*51378*	*34978*	*13569*	*16074*	*15049*
total genes	*496087*	*397436*	*34588*	*35748*	*35748*

Differential expression analyses of the full-factorial experimental design were performed on expression data produced using four alternative mapping approaches (in addition to the main method. Numbers of significantly differentially expressed genes in edgeR general linear models (FDR < 0.05) due to seasonal environment, food stress treatment, family (genetic background), or their interactions are indicated for four alternative mapping approaches as well as the main method. The bottom two rows indicate total numbers of expressed genes after filtering for low expression, and total numbers of genes before filtering, respectively. See Methods and Supplementary Table 5 for descriptions of each approach.

Supplementary Table 2. Genes significantly affected by food stress analysed separately for each tissue for each seasonal environment

gene	stress effect	season	body part	Fold Change (^{2}log)	p value *)	Uniref90 protein name
evgtrinc269331_g1_i4	up	dry	abdomen	2.6	0.04936	Trypsin AiT9
evgtrinc273562_g2_i1	up	dry	abdomen	1.5	0.04936	Beta-fructofuranosidase 2
evgtrinc281022_g4_i1	up	dry	abdomen	1.1	0.00898	Sugar transporter 12
evgtrinc279897_g1_i2	up	dry	both	2.3	0.01768	NA
evgtrinc288360_g3_i1	up	dry	thorax	2.0	0.03438	NA
evgtrinc287818_g3_i2	up	dry	thorax	1.9	0.03438	NA
evgtrinc289482_g5_i1	up	wet	abdomen	4.0	0.00084	NA
evgtrinc290184_g2_i2	up	wet	abdomen	2.4	0.01513	Uncharacterized protein
evgtrinc284646_g1_i3	up	wet	abdomen	2.4	0.03299	NA
evgtrinc286859_g1_i4	up	wet	abdomen	2.4	0.03800	p260
evgtrinc244065_g1_i1	up	wet	abdomen	1.9	0.02135	NA
evgtrinc286931_g1_i3	up	wet	abdomen	0.7	0.03299	Putative reverse transcriptase
evgtrinc278939_g1_i1	up	wet	abdomen	0.5	0.01954	Putative uncharacterized protein
evgtrinc283258_g1_i1	down	both	abdomen	1.8	0.00003	Uncharacterized protein
evgtrinc212398_g1_i1	down	both	abdomen	1.3	0.00006	Bombyrin
evgtrinc288751_g1_i1	down	dry	thorax	1.1	0.03438	Putative monocarboxylate transporter
evgtrinc401477_g1_i1	down	wet	abdomen	1.9	0.02518	NA
evgtrinc274166_g1_i1	down	wet	abdomen	1.7	< 0.00001	Uncharacterized protein

evgtrinc286831_g2_i1	down	wet	abdomen	1.4	< 0.00001	Moderately methionine rich storage protein
evgtrinc281179_g1_i1	down	wet	abdomen	1.3	0.00010	Methionine-rich storage protein
evgtrinc286831_g3_i2	down	wet	abdomen	1.2	0.00029	Moderately methionine rich storage protein
evgtrinc287884_g3_i1	down	wet	abdomen	0.9	0.00322	Arylphorin-type storage protein

* Corrected for multiple testing using Benjamini and Hochberg's multiple comparisons correction (false discovery rate).

Supplementary Table 3. Summary of sample sizes per tissue per experimental factor as used in edgeR general linear models

Factor	Number of groups compared	Number of individuals per group per tissue
Seasonal environment	2 (wet vs. dry)	36
Food treatment	2 (control vs. stress)	36
Family	7 (families 1 through 7)	8 or 12
Season by family	14	4 or 6
Food by family	14	4 or 6
Season by food	4	18
Season by food by family	28	2 or 3

Analyses for abdomen and thorax were performed separately. See Supplementary Data 4 for per-individual treatment group membership.

Supplementary Table 4. High completeness of assembled contigs in *de novo* transcriptome assembly

expected protein length (AA)	number of contigs per OHR category									
	**0–0.1**	**0.11 – 0.2**	**0.21 – 0.3**	**0.31 – 0.4**	**0.41 – 0.5**	**0.51 – 0.6**	**0.61 – 0.7**	**0.71 – 0.8**	**0.81 – 0.9**	**0.91–1**
0–100	0	0	3	8	29	20	36	49	68	190
101–200	0	13	24	49	89	110	169	208	284	922
201–500	8	50	104	175	224	338	333	372	575	2907
501–1000	10	40	87	154	249	298	234	224	239	1044
1001–2000	14	39	56	114	115	114	77	60	64	198
2001–5000	3	18	23	21	32	22	14	5	9	17
5001–10000	0	0	0	4	0	0	0	1	0	0
>10001	0	0	2	0	1	0	0	0	0	0
Sum	35	160	299	525	739	902	863	919	1239	5278

Table shows the distribution of contig Overlap Hit Ratio (OHR) values, sorted by OHR value (top row) and expected protein length (first open column). See Supplementary Fig. 16 for a histogram.

Supplementary Table 5. Alternative transcriptome filtering mapping and transcript abundance estimation approaches

Approach	Transcriptome filtering	Mapping program	Transcript abundance estimation	Transcript feature
alternative 1	full Trinity assembly	Bowtie2	RSEM	isoform
alternative 2	full Trinity assembly	Bowtie2	RSEM	gene
alternative 3	Evigene-enriched assembly	Bowtie2	RSEM	gene
alternative 4	Evigene-enriched assembly	NextGenMapper	SAMtools idxstats	gene
main method	Evigene-enriched assembly	Bowtie2	SAMtools idxstats	gene

Four alternative approaches (in addition to the main method) were used to produce expression data from raw RNA-seq reads, varying transcriptome filtering, mapping program, transcript abundance estimation, and transcript feature level. See Supplementary Table 1 and Supplementary Fig. 2 for results of each approach.

Supplementary References

1 Tajima, F. Statistical method for testing the neutral mutation hypothesis by DNA polymorphism. *Genetics* **123**, 585-595 (1989).

2 Clutton-Brock, T. H. Reproductive Effort and Terminal Investment in Iteroparous Animals. *The American Naturalist* **123**, 212-229, doi:10.1086/284198 (1984).

3 Harris, I., Jones, P. D., Osborn, T. J. & Lister, D. H. Updated high-resolution grids of monthly climatic observations – the CRU TS3.10 Dataset. *International Journal of Climatology* **34**, 623-642, doi:10.1002/joc.3711 (2014).
