# Supplementary material for: Strong phenotypic plasticity limits potential for evolutionary responses to climate change
Source: Nat Commun. 2018 Mar 8;9:1005. doi: 10.1038/s41467-018-03384-9 (PMC5843647; doi:10.1038/s41467-018-03384-9)
Supplement: Supplementary file 2 — Description of Additional Supplementary Files [file 41467_2018_3384_MOESM2_ESM.pdf]

Description of Additional Supplementary Files

File Name: Supplementary Data 1
Description: **All season-biased genes, i.e. genes showing a significant effect (FDR < 0.05) of the seasonal environment in thorax, abdomen, or both.** For each gene, the following information is provided: season bias (log_2 fold change), average expression (log_2 CPM), p value for the effect of the seasonal environment (fdr corrected), UniRef90 protein ID, Uniref90 protein name, and Uniref90 best hit organism.

File Name: Supplementary Data 2
Description: **Gene Set Enrichment analyses for the systemic, abdomen-specific and thorax-specific plasticity programme, as well as discordant genes showing opposite patterns of season bias between the two body parts.** See Supplementary Figure 8 for a visualisation and grouping of these terms, and Supplementary Data 1 for full lists of season-biased genes.

File Name: Supplementary Data 3
Description: **Genes showing a significant effect (FDR < 0.05) of the interaction between seasonal environment and family (i.e. genotype-by-environment interaction) in thorax, abdomen, or both.**

File Name: Supplementary Data 4
Description: **Sequencing information and experimental treatment group information for all 144 sequenced RNA-seq libraries** (72 individuals, abdomen and thorax was sequenced for each individual).
